# “The significance of context”: Fit, friction, and function in the work lives of early career academics with ADHD

**DOI:** 10.1371/journal.pone.0361208

**Published:** 2026-10-29

**Authors:** Lorna G. Hamilton, Anusha V. Ramji, Mahmoud Elsherif, Steven K. Kapp, Justin Sulik

**Affiliations:** 1 School of Education, Language and Psychology, York St John University, York, United Kingdom; 2 Section of Pediatric Population Science, Outcomes, and Disparities Research, Division of Pediatric Hematology/Oncology, Rutgers Cancer Institute, New Brunswick, New Jersey, United States of America; 3 Psychosis Studies Department, Institute of Psychiatry, Psychology & Neuroscience (IoPPN), King’s College London, London, United Kingdom; 4 Department of Psychology, University of Birmingham, Birmingham, United Kingdom; 5 Department of Psychology and Vision Sciences, University of Leicester, Leicester, United Kingdom; 6 Centre for Interaction, Development, and Diversity, School of Psychology, Sport and Health Sciences, University of Portsmouth, Portsmouth, United Kingdom; 7 Cognition, Values & Behaviour Lab, Munich Interactive Intelligence Initiative, LMU Munich, Munich, Germany; Nagoya University: Nagoya Daigaku, JAPAN

## Abstract

Attention deficit hyperactivity disorder (ADHD) is associated with various traits that influence individuals’ experiences at work. While some may be experienced as challenging (e.g., when differences in executive function impact time management), others can be more positive (e.g., when curiosity motivates exploration and discovery). Plausibly, academia is a work environment where these positive aspects should be valued. However, ADHD is still often viewed from a medicalised perspective in workplace settings, despite the neurodiversity paradigm offering an alternative, non-pathologising way to frame these individual differences. Research has begun to explore employment experiences and outcomes for adults with ADHD; however, there is currently little data relevant to academic workplaces specifically. This qualitative study offers a critical realist analysis of interviews with early-career academics, seeking to understand first-person ADHD perspectives on establishing an academic career. Consistent with the neurodiversity paradigm, the analysis foregrounds (mis-)matches between individual ‘neurotypes’ and workplace environments. It also highlights an ecological view by exploring how ADHD matters both for individual careers and for collective knowledge production. Findings suggest that academia can, in principle, be a rewarding environment for ADHDers, though social and institutional pressures often inhibit this potential. Furthermore, having an ‘ADHD neurotype’ influences both individual career trajectories and wellbeing at work; at the same time, knowledge production at the collective level may benefit from the inclusion of ADHD academics. The findings are discussed in reference to ecological models of neurodiversity and their implications for universities aiming to facilitate neurodivergent contributions and inclusion at work.

## Introduction

Meaningful employment is a reliable determinant of quality of life [1]. However, despite growing societal awareness of the potential benefits of cognitive diversity in the workforce, neurodivergent people continue to encounter barriers in education, healthcare and workplace settings that limit career trajectories [2]. Academic careers are often theorised to be appealing to (some) neurodivergent people, due in part to opportunities for deep, focused study of topics of intrinsic interest. Though robust prevalence data is lacking, surveys suggest that autism, attention deficit hyperactivity disorder (ADHD), and other forms of neurodivergence are substantially overrepresented in research organisations [3]. Nonetheless, university workplaces are generally not designed with neuro-inclusion in mind, which can precipitate isolation and burnout for individuals: ultimately, talented researchers leave the sector [4]. In this qualitative study, we analyse interviews conducted with early-career researchers with ADHD, with the aim of identifying neurotype–environment interactions that are potentially influential in the process of establishing an academic career.

There are diverse, sometimes conflicting conceptualisations of ADHD [5]. ADHD is clinically defined in terms of inattention, hyperactivity, and impulsivity symptoms. Here, we adopt a conceptualisation more consistent with the neurodiversity paradigm and the lived experiences of many ADHDers: framing ADHD in terms of ‘difference’ rather than ‘deficit’; resisting purely neurological accounts by highlighting personal experiences (which often reflect brain/biological differences without being fully reducible to those); expecting these experiences to be somewhat fluid across contexts; highlighting strengths as well as challenges; and situating both strengths and challenges (including disability) in person–environment interactions [6]. Accordingly, by “ADHD neurotype,” we mean both cognitive traits and subjective experiences that are common among ADHDers (including cognitive dynamism, adventurousness, and the ability to hyperfocus on topics of interest [7]), without assuming that there is a necessary and sufficient set of these traits or experiences that must be universal among (or unique to) ADHDers.

These contrasting conceptualisations give rise to some tension in language use [5]: we consulted our participants about preferred terminology and use “person with ADHD” and “ADHDer” interchangeably through the manuscript in line with their preferences. Non-pathologising terminology in relation to ADHD is not yet standardised, and we acknowledge that these language choices will not be right for everyone.

Recent literature has begun to examine the experiences of people with ADHD across various work environments, highlighting the importance of contextual fit in determining job satisfaction and career progression [8]. However, two open problems remain in applying insights from these studies to academic careers. First, academia is unlike many workplaces, both in practical terms (e.g., relative time flexibility; diversity of roles performed by a single individual) and intellectual terms (e.g., potential for discovery or complex problem-solving). Second, much research has focused only on individual-level factors, chiefly cognitive traits associated with ADHD, that may increase or decrease the likelihood of ‘success’ in the workplace [9,10]. In contrast, recent developments in the philosophy of neurodiversity offer an ecological view of functioning [11], which goes beyond reframing apparent individual deficits as strengths (a strategy that still maintains an individual-level focus) by considering collective-level outcomes in specific environmental contexts (e.g., a university; the scientific community). In this study, we contextualise ADHD experiences within an ecological view of academia.

First, though, we note that the term “functioning” will occur in several senses here. These are conceptually distinguishable but frequently overlap in our interpretation of participants’ experiences, hence our deliberately holistic approach to this term. Depending on context, it can variously refer to:.

- Cognitive functioning: differences in basic cognitive processes including executive function, attention, and memory.
- Everyday functioning: differences in managing or navigating the demands of daily life (often—but not necessarily—related to cognitive functioning) with implications for subjective wellbeing.
- Professional functioning: differences in cognitive/everyday functioning applied to a work setting (here, an academic setting, with implications for research productivity, teaching, and other professional activities).
- Collective functioning: broader group-level patterns in scientific progress, where this is conceived of as an inherently social enterprise with sometimes counterintuitive mappings between individual performance and collective outcomes.

### ADHD in the workplace

Relative to neurotypical peers, ADHDers experience lower employment rates and performance ratings, alongside higher rates of underemployment and job turnover [12,13]. Explanations of these patterns appeal to psychological factors (e.g., executive function and attention regulation ‘deficits’), which are assumed to underlie observable difficulties with task initiation or completion, time management, organisation, and emotion regulation [9]. However, neurodiversity-informed explanations also consider potential (mis-)matches between individual profiles and environmental demands [14]. While roles that require repetitive, sustained focus or significant bureaucratic navigation can increase stress and reduce productivity for ADHDers, workplace environments offering structure, clear deadlines, or immediate feedback mitigate differences in attention regulation characteristic of ADHD [15,16]. Conversely, environments offering novelty and opportunities for curiosity-driven problem-solving can align well with ADHDers’ often-reported preference for high stimulation or interest-driven motivation [8,17].

Given inequalities in employment outcomes, it is essential to understand first-person ADHDer perspectives, including experiences with contextual factors that enable or undermine effective ‘functioning’ in the workplace. We note that cognitive profiles are highly heterogeneous in ADHD, especially given high levels of co-occurrence with other neurodevelopmental conditions [18], just as different workplaces provide unique ecological niches. We therefore aim to identify unifying factors that may explain the diverse experiences of academics with ADHD.

### Academia as a work environment

Here, we focus on select aspects of the academic work environment that we expect to be relevant to ADHDers, drawing on existing literature on ADHD and employment, autobiographical accounts, and some of our experiences as neurodivergent academics. Academic work is relatively flexible, allowing for some agency in choosing one’s work times and tasks. However, academics typically have little control over the overall *amount* of work they do [19]; this combination of flexibility and high workloads can negatively affect wellbeing and family life [20]. Flexibility may also be a symptom of precarity or casualisation, particularly for early-career researchers [21]. Thus, though flexible working patterns are often recommended for neuro-inclusion in work environments, successfully navigating that flexibility in the context of academia may bring its own set of challenges for ADHDers.

Academic work is also ‘unbounded’ [22]: academics typically juggle numerous tasks, taking on disparate roles in research, teaching, public engagement, and citizenship without externally imposed limits. Beyond ‘work–life balance’, this raises issues of ‘work–work balance’ in the struggle to meet multiple, competing demands, which may further compromise the benefits of flexibility [23]. The challenges of unbounded work put additional pressure on time management, planning, and attention switching, which are common challenges for ADHDers [24,25]. On the other hand, academic work is often characterised by novelty (new ideas, projects, collaborations) and typically offers scope for intellectual curiosity. Since the ADHD neurotype is characterised as novelty-seeking and curiosity-driven [26], the intrinsic rewards of academic work may help offset workplace challenges.

### Academia as an ecology

The issues raised so far are consistent with a neurodiversity perspective, defocusing individual successes and failures in order to highlight behaviour–environment (mis-)matches. However, an ecological perspective also contextualises individual behaviours in terms of group-level functioning [11]. For instance, a standard neurodiversity-informed perspective might argue that ADHD attention patterns can sometimes lead to enhanced focus or creativity, depending on the nature of one’s current task. This conceptualisation might prompt efforts to improve the work environment and, consequently, support these strengths or facilitate individual flourishing. An ecological view goes further, by distinguishing individual- and collective-level outcomes and by considering ‘function’ in light of both.

To illustrate: if a PhD student with ADHD is struggling to focus on a literature review task for their dissertation and instead gets side-tracked by diving into several new (but task-irrelevant) topics, this may have various consequences for that individual. The student may thoroughly enjoy this ‘side quest’; they may also publish less overall due to time spent on scattershot reading. They may think that the former intrinsic reward outweighs the latter cost, even if it has detrimental knock-on effects on their career. A standard neurodiversity-informed approach might reframe an apparent distractibility ‘deficit’ as intrinsic curiosity, or might find ways to mitigate negative effects on the individual (e.g., suggesting how improved office design might minimise distractibility, or providing institutional support to counterbalance its adverse career impacts). By contrast, an ecological view acknowledges that this behaviour may yield detrimental outcomes for the individual while benefitting the collective: it might advance scientific progress overall if some researchers explore in this way, even if it negatively influences individual research careers. Indeed, epistemic progress involves a trade-off between pursuing whatever seems the current-best strategy and exploring new areas, such that it is better for science if a minority are not directly aiming at whatever the broader community currently deems best [27,28].

In this qualitative study, we analysed first-person accounts of early-career academics with ADHD working across diverse disciplinary, institutional, and national contexts. We aimed to characterise commonalities in neurotype-environment (mis-)matches and to identify potential explanatory factors underpinning both individual career trajectories and collective functioning within academic ecosystems (e.g., research groups). Since first-person perspectives of neurodivergent people in academia are only recently emerging in the research literature, this study addresses the pressing need for more ‘insider’ research to reframe ‘the problem of ADHD’ in the workplace [29]. Thus, one of the aims of this study is to explore how ADHD academics’ perspectives on their own experiences (whether strengths or challenges) at the individual level might inform theorising about ADHD contributions to academia at the collective level.

## Method

### Research paradigm and positionality

In this study, we adopt a neurodiversity-informed ontology of ADHD, conceptualising traits that are often framed as deficient in relation to an idealised ‘norm’ as valuable human diversity [30]. While features of ADHD can be experienced as disabling or distressing for individuals in the context of societal expectations, practices, and institutions (e.g., education, employment), we do not conceptualise these genetically-influenced traits as inherently pathological.

The authorship team includes researchers with experience of navigating academic careers as ADHDers; some of us are therefore positioned as ‘insider researchers’. Bertilsdotter Rosqvist et al. [29] highlight the need for more insider research on ADHD to counteract the historic exclusion of neurodivergent people from knowledge-generation processes that directly affect their lives (i.e., epistemic injustice) [31]. Insider research can correct dominant narratives of human difference that are overly biased towards deficit-focused explanations [32]. In this respect, ADHD research lags behind the participatory turn in autism studies, remaining primarily examined through a medical-psychiatric lens [33]. Recent reframings emphasise how neurodivergent individuals offer valuable insights to the study of neurodiversity, in part due to cognitive and motivational characteristics such as non-conformity and intellectual creativity [34]. On the other hand, insider research demands particularly careful and continual reflexivity to ensure that personal experiences do not unduly influence the interpretation of data or de-centre participants’ insights. We addressed this risk by maintaining a reflexive journal throughout the study, considering disconfirming cases and alternative explanations, surfacing commonalities and differences in perspectives among the research team in regular group meetings, and through iterative review of data coding and analysis by different members of the team. Finally, we ‘member checked’ our draft analysis. A link to the preprint was sent to all participants prior to journal submission with an invitation to feed back on our analysis, including use of their quotations. Seven participants provided feedback, which largely endorsed the analysis, and resulted in two amendments: (1) we provided more context to one data quotation; (2) we integrated the impact of systemic workloads in our analysis of the burnout cycle.

### Ethics statement

Primary ethical approval for the study was obtained from the School of Education, Language and Psychology Ethics Committee at York St John University (ETH2223−0023). Participants provided fully informed written consent before taking part in interviews.

### Participants

We aimed to recruit early-career academics (defined as ranging from PhD study to 10 years post-PhD) with ADHD. Given widespread barriers to clinical ADHD assessment [35,36], formal diagnosis and self-identification were equally acceptable. We targeted academics working at a university who were sufficiently fluent to participate in an interview conducted in English. Recruitment advertisements were posted in online academic and neurodiversity networks and on social media. Participant recruitment took place between May and August 2023. Recruitment combined features of purposive sampling (by design, we targeted early-career academics with ADHD) and convenience sampling (we recruited by advertising in our established professional networks and on social media).

In total, 20 participants residing in 10 different countries participated in the study. We note that "sample size justification" is an ill-defined term in qualitative research, and judge the adequacy of our data by inherently subjective features such as richness and depth. (For further discussion, see SM1.) Participant demographics are presented in Table 1. Seventeen participants had a clinical diagnosis of ADHD, mostly received in adulthood. The mean age of diagnosis was 28 years (*SD* = 9.00). Participants volunteered to take part in the study without compensation.

**Table 1:** Participant characteristics.

Demographic factor	Category	n (%)
Gender	Woman	11 (55%)
	Man	8 (40%)
	Non-binary	1 (5%)
Ethnicity	White	17 (85%)
	Racialised Asian	1 (5%)
	Racialised Black	1 (5%)
	Mixed heritage	1 (5%)
Co-occurring neurodivergence/ mental health condition (*including anxiety, depression, OCD, autism, dyspraxia*)	Yes	13 (65%)
	No	7 (35%)
Current role	PhD/ doctoral student	10 (50%)
	Postdoctoral research role	2 (10%)
	Lecturer/ senior lecturer/ assistant professor	6 (30%)
	Lab technician	1 (5%)
	Clinician and adjunct	1 (5%)
Subject specialisation	Psychology	11 (55%)
	Education	3 (15%)
	Neuroscience and biological sciences	3 (15%)
	Social sciences	2 (10%)
	Speech sciences	1 (5%)
Country of residence	United Kingdom	5 (25%)
	United States of America	3 (15%)
	Canada	3 (15%)
	Australia	2 (10%)
	Belgium	1 (5%)
	France	1 (5%)
	Israel	1 (5%)
	Netherlands	1 (5%)
	Spain	1 (5%)
	Sweden	1 (5%)

Participants completed a brief survey on their language preferences concerning ADHD and neurodiversity (adapted from Keating et al. [37], available from the project’s repository in the Open Science Framework at https://osf.io/hb32d/). Attention Deficit Hyperactivity Disorder (ADHD) was the preferred term of 75% of the sample, over alternatives including Attention Deficit Hyperactivity Condition (ADHC) and Variable Attention Stimulus Trait (VAST). ‘Person with ADHD’ was preferred over ‘ADHDer’ by 95% of participants, although both were deemed acceptable. The majority of participants (60%) preferred ADHD to be conceptualised as a neurological/brain difference, rather than a disorder or mental health condition. These conceptual and terminology preferences are adhered to throughout the paper.

### Data collection

We conducted semi-structured interviews via video-conferencing software (Zoom or Microsoft Teams). Semi-structured interviews balance consistency with the flexibility to explore individual experiences and perspectives in depth [38]. The interview schedule (available at https://osf.io/hb32d/) comprised a series of open questions, with optional follow-up prompts, relating to participants’ career trajectories, motivations and values as researchers, academic practices and institutional culture, and perceptions of the impact of ADHD on their academic work. Questions were developed in consultation with a group of neurodivergent early-career academics outside the core research team. Participants were offered access to the interview schedule in advance of their interview.

Two pilot interviews were conducted prior to the study. These interviews were recorded, viewed, and discussed among the research team to refine the question schedule and agree on common principles, e.g., active listening, avoiding ambiguous or multi-component questions, combining closed and open follow-up prompts to accommodate diverse communication preferences, allowing processing time, and offering breaks [39]. Four members of the research team interviewed the 20 participants, with interviews lasting between 41 and 110 minutes (*M* = 65.95, *SD* = 17.61). The record and transcribe functions in Zoom and Teams were used to generate initial transcripts, which were subsequently amended for accuracy and formatted according to an agreed set of orthographic transcription conventions. The complete set of transcripts was then combined, read, and checked across the whole research team. Interview transcripts cannot be made openly available as this is a qualitative study drawing on data that (a) covers sensitive subject matter; (b) carries a high risk of identification of participants due to the specific nature of the sample (early-career researchers with ADHD); (c) by design, reflects personal experiences throughout.

### Data analysis

We adopted a critical realist approach to thematically analyse the interview data [40,41]. This emerging analytic framework builds on the methodological foundations of reflexive thematic analysis developed by Braun and Clarke [42,43], both in acknowledging the inseparability of researcher subjectivity from knowledge production and in several steps of the analytic process. However, realist ontology assumes a real, objective level of existence of things in the world, albeit one that cannot be directly measured without filtering through individual human minds, language, and culture [44]. Critical realism provides a unifying framework for quantitative and qualitative research that seeks to identify causal explanations of social phenomena. Multiple causal mechanisms interact in complex ways to determine life trajectories, and in-depth qualitative analysis is well-placed to identify and describe these mechanisms, albeit imperfectly as “causal tendencies” [40], referring to the *potential* for a factor to exert an effect, based on its own properties and its relations with other factors. It has been argued that critical realism is a particularly well-suited philosophical framework for social justice-oriented neurodiversity research [45,46].

We used the five analytic steps proposed by Fryer [40] as a guide for the current analysis. The process was not linear, involving frequent circling back to previous steps to refine the analysis and evaluate its empirical adequacy and explanatory power [41].

(1) We developed a set of research questions before collecting the data, which evolved as the analysis progressed. The final questions, addressed by the analysis presented here, are explanatory in nature:
  RQ1: How do neurotype–environment (mis-)matches characterise the work experiences of early career researchers with ADHD?
  RQ2: How does an ADHD neurotype contribute to individual and collective functioning within academic ecosystems?
(2) All authors familiarised themselves with the dataset by reading the transcripts, noting and then collating initial thoughts in a shared document.
(3) Inductive, descriptive coding was applied to the entire dataset by Authors 1 and 2 separately, who then met to compare ideas and approach consensus on the initial coding scheme. Codes were both semantic and latent. Subsequently, Author 1 recursively reviewed the coding scheme by collapsing similar codes, broadly grouping them in an ecological framework according to whether they related to ‘*intrinsic’ features of ADHD*, *person-environment fit in academia,* or *lifespan trajectories*; the interpretative validity of the developing coding scheme was checked iteratively by the other authors and revisions were suggested as needed. In Fryer’s analytic approach, data are analogous to the critical realist concept of *experiences* (i.e., what is perceived, felt, or observed by individuals), while codes relate to *events* (i.e., causally generated occurrences in the world, some of which are experienced by individuals). In the latter stages, codes were selectively aggregated in a more interpretative analysis, beginning to identify potential underlying *structures* (i.e., generative mechanisms, or causal tendencies, that produce events and experiences).
(4) Initial themes were developed to capture causal tendencies that could address the research questions, accounting for the diverse experiences recounted by participants. We used retroductive reasoning, i.e., the process of working back from data to generate potential explanations for those data through hidden, underlying mechanisms. In doing this, we drew on aggregated data from salient codes (including participants’ own explanations), relevant theoretical knowledge (notably ecological functions accounts of neurodiversity), and the subjective experiences and insights of the research team, to refine a set of *explanatory themes*. We assessed the explanatory power of the analysis by revisiting the data, identifying disconfirming cases, and amending the thematic structure accordingly.
(5) In the final step, we linked our thematic structure to the broader literature on ADHD/ neurodivergence in workplace settings, then generated and reviewed conclusions from the analysis.

A reporting checklist, summarising these and other design decisions, appears as S1 File; the project’s online repository (https://osf.io/hb32d/) contains data files that track the evolution of codes from Step 3 above.

## Results

Our analysis resulted in two themes, defined by six subthemes (Fig 1). ‘Neurotype environment fit and friction’ identifies (mis-)matches between individual traits and features of academic workplace environments that influence the early-career experiences of ADHDers. ‘ADHD and academic functioning’ captures ways in which an ADHD neurotype influences both individual and collective functioning in academic workplaces.

**Fig 1:**
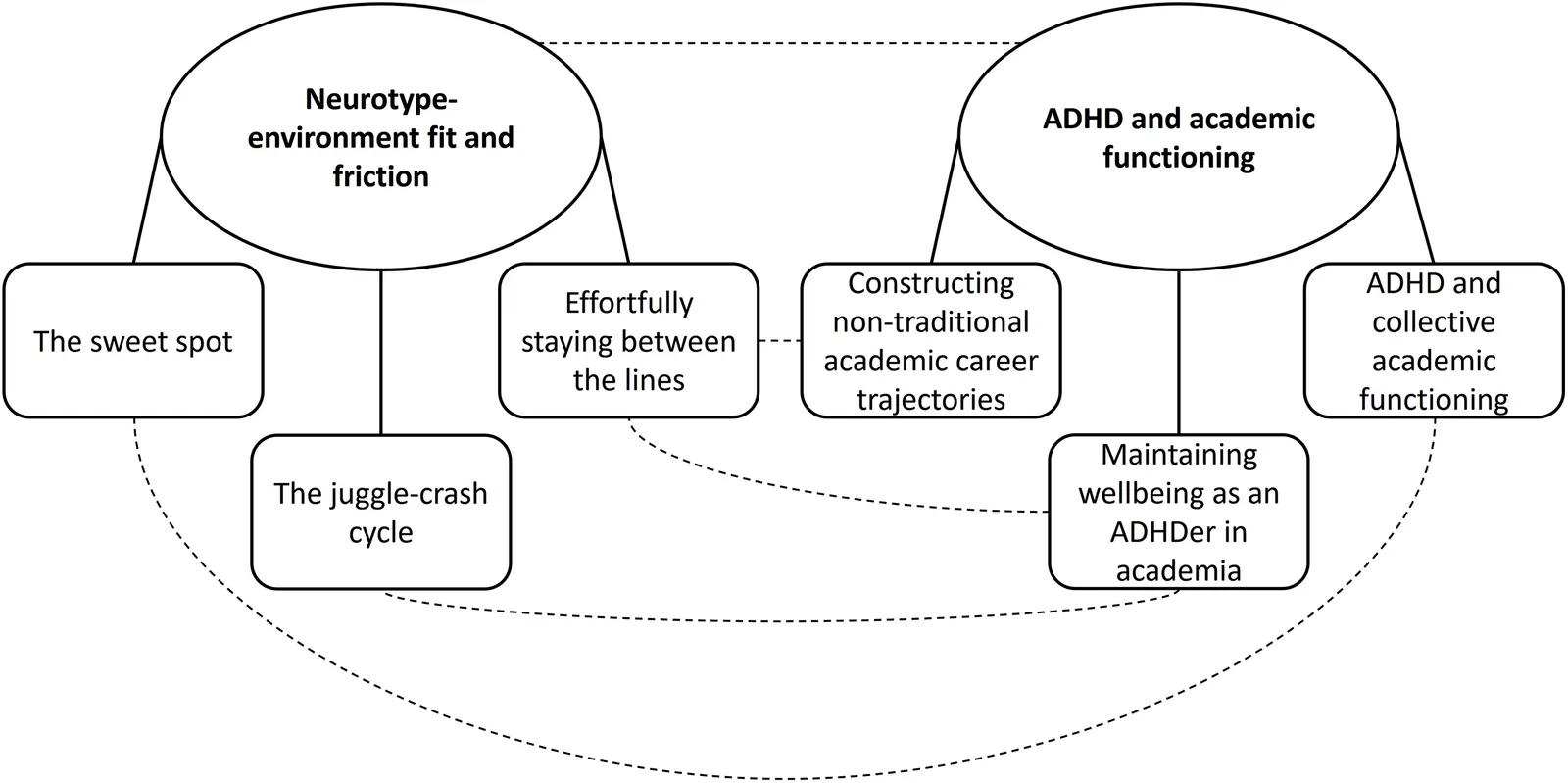
Thematic map:. The interplay between neurotype-environment fit and friction (left) and the impact of ADHD on academic functioning (right), highlighting the relationship between individual experiences (e.g., the juggle-crash cycle) and broader academic trajectories.

### Theme 1: Neurotype-environment fit and friction

The participants’ accounts depict a complex interplay between heterogeneous, intrinsic features of ADHD and aspects of academic working environments. This interplay encompasses both fit (*1a: The sweet spot*) and friction (*1b: The juggle-crash cycle; 1c: Effortfully staying between the lines*).

#### 1a. The sweet spot: Where academic work life fits an ADHD neurotype.

*I feel like academia almost creates an ADHD-like phenotype sometimes. It’s just the environment we’re in, where we’re constantly bombarded with all this information, there’s so many expectations. It kind of makes me feel at home.* (Non-binary person, 31, Canada)

Participants noted aspects of their jobs that enabled them to work in synchrony with their perceived ‘intrinsic’ ways of being in the world. Descriptions of personal characteristics reflected both clinical and strength-based conceptualisations of ADHD: dysregulated attention, unpredictable executive functioning capacity or energy cycles, a preference for novelty over sameness, creativity and innovation, and, for some, an intuitive ability to make effective decisions under pressure. Participants often described themselves as curiosity-driven and able to focus intensely on subjects of interest, facilitating in-depth research activity:

*Being able to hyperfocus on something that I really like, or when I have a statistical problem, I will not go to sleep until I’ve solved it*. (Woman, 40, UK)

*Research is unpredictable and it’s alive and I really enjoy that challenge*. (Woman, 39, France)

For ADHDers who perceived themselves as often being judged by others as “*too much”,* the academic environment allowed them to openly pursue their intense inquisitiveness and ‘unmask’ passionate enthusiasm for a subject in a way that felt socially acceptable. Beyond research, the variety of tasks and responsibilities attached to most academic roles could suit the tendency to seek novelty and become bored quickly. While individuals differed in the aspects of their roles that they found rewarding, a common appreciation for the variety and stimulation of academic life was expressed across the interviews.

*Academia is super multifaceted. It’s teaching and it’s research and it’s supervision and mentorship and writing and reading. If I get bored, there’s always something else for me to jump to. I always have way too many tabs and way too many documents open on my desktop. But on some level, I think that serves me well in academia, as long as things don’t fall through the cracks. So I actually feel like because of my ADHD, I’m able to succeed in my current position*. (Man, 42, Israel)

Participants talked about the “*buzz”* of sharing passions through teaching, and the fun of engaging students in the classroom. Storytelling, clarity of explanation, and spontaneity were commonly identified as pedagogical strengths. Past difficult experiences of education meant that they were closely attuned to diverse student needs, and ready to adapt their teaching practice in response to student behaviours that others might misinterpret as ‘difficult’:

*If there are neurodivergent people in the class, sometimes they’ll need to ask questions straight away and it seems like they keep asking, asking. But if they don’t, they’re gonna sit there for an hour not taking anything in. If they get that question out and I’ve answered it, then they can start taking in stuff again*. (Man, 43, UK)

Beyond workload variety, the relative autonomy and flexibility of academic life were often framed as benefits for ADHDers. Participants appreciated the capacity to organise work tasks to align with their energy patterns. Often this entailed working at unusual times, mixing on-campus time with work in other spaces, and intense periods of productivity interspersed with times where focus was more diffuse.

*I can choose when I lesson-plan, whenever I feel like it. If it’s two o’clock in the morning or 8 a.m. or whatever else. So that feels like a very ADHD thing of not having prescribed times necessarily, that my brain is working at its best*. (Woman, 34, Canada)

Optimally, then, the academic ecosystem could provide a fitting context for ADHDers to work with, rather than against, their differences. This ‘goodness-of-fit’ in the university context was often contrasted with more negative experiences in school or other employment settings.

#### 1b. The juggle-crash cycle.

*When I feel very engaged, I send emails for new collaborations and new projects, especially late at night when you feel like you’re really active. But then a week after, you can feel very burned out and then I regret all the things I said “yes” to. Yeah, it goes in cycles*. (Woman, 24, Belgium)

The flipside was that people tended to over-commit, initiating and/or accepting invitations to work on any project that interested them. This could lead to a repeating pattern of peaks of excitement and motivation followed by troughs of overwork and burnout. On the one hand, juggling multiple projects and commitments could sustain interest and motivation at work. To colleagues, this tendency to multi-task could give the impression of high competence and productivity, thus bolstering ADHDers’ confidence that they were ‘doing academia right’.

*My usual thing was when I was in the lab, I would do three experiments at once and I just loved that. You know just being super busy and juggling all of the things […]. Everyone was just looking at me, like “What the heck is she doing*?” (Woman, 30, UK)

On the other hand, very high levels of activity were often unsustainable, precipitating periods of disengagement or burnout. Participants were highly aware of fluctuations in their ability to keep up with workplace demands, which could be exacerbated by the flexible, unbounded nature of academic work.

*It’s a weird balance of like, “I want to do all of the things, and yet I do not feel capable of doing all of the things”. When I am feeling my best, I am […] like a powerhouse, I can do magic. And then other times my brain just goes [raspberry sounds], I do not want to do any of that*. (Woman, 34, Canada)

The experience of unpredictable capacity, motivation, and focus at work was experienced as a stressor, fuelling anxiety and doubts about one’s place in the academy. Task prioritisation and project completion were acknowledged as significant challenges. Managing multiple, complex tasks and competing deadlines within a relatively unstructured work environment sometimes led to paralysis, which could cause considerable distress.

*My anxiety was so high I couldn’t work. […] It felt a bit like I was frozen. And then I had deadlines coming up and all of a sudden I was so stressed, I was working day and night to finish it, you know? I did get it finished and it was fine, but it was so exhausting. It was […] a terrible experience*. (Woman, 30, UK)

Cumulative experiences of this ‘juggle-crash’ cycle over time meant that people could doubt their capabilities and judgement, and even avoid opportunities at work, anticipating that they would result in failure.

*There’s still this little bit in my head that is like, “Well, don’t get too excited. Yes, these things would be cool. […] But you don’t have a good track record on following through”, and it has made me a bit nervous about doing these things, ‘cos on the one hand [there’s] this intense excitement about, “Oh my God, we could do this and we could do this” […]. And on the other hand, the bit of me that intensely remembers not being able to do those things in the past, which is why I said “slightly traumatised”, because it does feel almost like a bruise*. (Man, 36, UK)

In summary, the unbounded array of projects, tasks, and collaborations that early-career researchers can embark upon, in combination with a tendency toward impulsive decision-making and unpredictable motivational energy, commonly led to over-work, stress, and related mental health challenges among participants.

#### 1c. Effortfully staying between the lines.

*“You’re running in place just to stay in it.”* (Man, 36, UK)

Participants identified how rigid university processes, structures, and traditions often do not accommodate neurodivergent differences. They were cognisant of the tension between the originality of thought required in academic research and teaching on the one hand, and the requirement to comply with fixed hierarchical roles and structures on the other. Many were critical of systems that seemed to be adhered to for tradition’s sake, rather than being designed for optimal outcomes or greater inclusion.

*If you’re [an academic leader] who really wants to change things — which I think is what I would want to do […] and they’re like, “No, no, we’ve done it this way for a long time. We can’t do that.” I think it would be quite frustrating*. (Man, 43, UK)

There was a common perception that creeping managerialism in the academy had led to a proliferating administrative burden that did not play to ADHD strengths. This was sometimes voiced as a concern that divergent thinking is becoming less valued in contemporary academia. Finding workarounds for administrative tasks absorbed valuable time and energy; people felt that they were often working harder than others for the same outcome, drawing on compensation techniques developed over many years. Complex and time-consuming administrative tasks could also disadvantage people via the so-called ‘ADHD tax’ [47], whereby core difficulties in task initiation, working memory, organisation, or impulsivity result in financial loss. This was particularly challenging for postgraduate students and early career researchers on precarious contracts, who described how forgetting to submit timesheets and other administrative paperwork impacted their financial wellbeing. Universities rarely provided support mechanisms to mitigate these neurotype-related risks.

*Every year I have to fill out a form, to literally just confirm that I’m still a student. And I have to send it back to some lady, and she gives me health insurance. And I forgot to do that a month ago, and then I found out that my health insurance had lapsed […]. I also just forgot to follow up about being reimbursed for a conference that I went to. And so I was out, like over 1000 dollars for just too long, because I didn’t follow up, I didn’t gather the receipts*. (Woman, 26, USA)

Several interviewees highlighted the discrepancy between the accommodations available to students and the lack of support available for employees. There was a common perception that, in the context of high workloads and an endless array of possible tasks and demands, inclusive practice for university employees becomes a peripheral concern, with contrasts drawn between the flexibility offered to students to manage assessment deadlines (e.g., a five-day extension offered to neurodivergent students) and the rigidity of timelines for administrative and marking deadlines for neurodivergent academic staff.

*If I told my manager I had ADHD and was struggling to complete the marking with the specified deadline, he’d just give my work to another lecturer. I wouldn’t get another five days to mark the work, would I? […] And sometimes I would like that extra five days, because sometimes it really is a battle. […] But I don’t wanna give more work to one of my colleagues, so I just have to plough through it*. (Man, 43, UK)

Institutional support for disabled staff was variously described as non-existent, generic, and unhelpful, or performative. Participants were therefore often reluctant to disclose neurodivergence, or to engage with support sessions that were perceived to be imposed from an outsider perspective, rather than co-created with neurodivergent people.

In summary, participants frequently experienced a tension between the criticality and innovation needed for academic work on the one hand, and resistance to change within inflexible academic systems and hierarchies on the other. They tended to expend a great deal of mental energy trying to comply with institutional expectations and systems. Universities were rarely perceived as inclusive of divergent thinking and processing styles, and people expended considerable effort trying to stay ‘between the lines’ – or mask their neurodivergent traits – to meet the (often implicit) demands of their workplace.

### Theme 2: The impact of ADHD on academic functioning

From an ecological function perspective, neurocognitive diversity would be expected to influence academic functioning at both the individual and collective level. Here, we focus on individual functioning in terms of academic career trajectories (2a) and personal wellbeing (2b), and collective functioning in terms of knowledge generation in research groups or academic fields (2c).

#### 2a. Constructing non-traditional academic career trajectories.

*But I think if you look at most academics’ careers, there’s a clear singular thread. Very clearly I’ve danced [between disciplines]. It’s been a ride!* (Man, 37, Netherlands)

Many of the participants combined multiple roles in their working lives (e.g., research and clinical practice; teaching and business consultancy) and several had joined academia later in life, having previously worked in other sectors. Novelty-seeking may make ‘patchwork careers’ more attractive, while difficulties with task completion and impulsive decision-making may make them more likely.

*I’m currently on the job market applying to tenure-track positions. I’ve had to make a narrative of all the work that I've been doing, even though it is a bit scattered. […] If I didn’t have ADHD, maybe I would have just stuck through with the training that I had and not tried to veer off into so many different other directions. But in a way, I feel like I’m happy with my trajectory and I think that I’m bringing something uncommon to the research that I’m doing*. (Non-binary person, 31, Canada)

The tendency to take on multiple projects, collaborations, or roles undoubtedly contributed to the *juggle-crash cycle* but also presented new opportunities and allowed people to make connections across disciplines, ideas, and perspectives. Methodological pluralism and interdisciplinary approaches to research were common among the participants.

*Basically, the more freedom the better […] when it comes to research. I don’t wanna delve into just one methodology. […] I don’t like limiting myself*. (Man, 28, USA)

This pluralistic approach to research could be perceived as diffuse or even chaotic by colleagues, particularly in scientific disciplines where programmatic research tends to be valorised. The impact of a novelty-seeking tendency on individual career paths depended on institutional cultures and national academic structures. In some contexts, people could construct a niche that allowed them to harness strengths and ‘succeed’ (e.g., successful grant capture supported by strong idea generation, leading to promotion; taking on leadership roles early in one’s career, and innovating for improvement). Conversely, other people experienced ADHD traits to be a barrier to academic ‘productivity’ and, therefore, career progression.

*I do think that ADHD might also be behind what makes me bad at academia. And a lot of my struggles, in terms of meeting deadlines, planning, and I guess getting distracted on things that I should be doing. And jumping between papers when I should just be finishing the one that is due in two days*. (Woman, 25, Australia)

Participants also recounted instances when inaccessible, and potentially discriminatory, institutional processes directly impacted career progression. For example, interview conventions could disadvantage people with neurodivergent communication styles that are unrelated to competence to do the job:

*Every time I've applied for their fellowship, I’ve got through to the interview stage. […] and I would get feedback that I need to have interview training. You know, “he struggled to stay on the topic. And he does lots of tangents and kept talking after addressing the question.” It just feels like that’s not fair. I’ve done a bunch of interview training. I know what I need to do. I know it’s good to be succinct and concise, and if I don’t care or if I’m very calm, you’ve caught me on exactly the right day, then I can. However, under usual interview conditions, when something really matters and you’re asking me to explain something about the project that I’m really excited about and I’m already nervous [I can’t]*. (Man, 36, UK)

Overall, while individual early career trajectories varied widely among the participants, there were common patterns linked to an ADHD neurotype, including non-linear, patchwork career paths and a pluralistic, ‘big-picture’ approach to research. Whether this led to career progression and/or job satisfaction depended on specific institutional and disciplinary contexts. In institutions where people were empowered to construct their own niche, they could harness their strengths and work collaboratively with others to compensate for difficulties.

#### 2b. Maintaining wellbeing as an ADHDer in academia.

*By the time I got the diagnosis, I was really drowning*. (Woman, 27, Australia)

Cumulative experiences of ‘not fitting in’ through the lifespan are commonly reported by neurodivergent people, with profound implications for mental health and quality of life [48]. This pattern was prevalent through the interviews, as the reported incidence of past or current mental health difficulties was very high, which impacted individual functioning in academia above and beyond intrinsic features of an ADHD neurotype.

*I’ve lived my entire life suffering from fatigue, and most of it is from managing my self-presentation and keeping up with everything*. (Man, 37, Netherlands)

Most participants discussed difficult experiences at school that had shaped their academic self-concept. Some described under-achieving academically in relation to ability and frequently being in trouble, with teachers often ascribing their learning-related behaviours and educational outcomes to laziness, lack of effort, or a bad attitude. In recounting a memory from school, one participant highlighted how education systems often prioritise the *performance* of attention, misinterpreting observable behaviours as indicators of inattention:

*There was this one time that I was in class and I was drawing on my notebook, but at the same time I was listening to my teacher. I was absolutely listening to one hundred per cent of what they’d said. But then they walked by me and they saw that I was drawing and they started reprimanding me. So I basically repeated back to them everything that they had just said to make them realise that I was paying attention and they stopped for a moment, looked at me and just said, “OK that’s fine, but please stop drawing.” So I stopped drawing and therefore I stopped paying attention*. (Man, 27, Spain)

For some, negative school experiences had ignited a desire to prove themselves competent by pursuing academic careers. For those who had received late diagnoses in particular, recognition of ADHD allowed them to reframe difficult past experiences in education and take a more compassionate view of what had previously been perceived as personal failings.

*Many people gave me this feedback […] “For a smart girl you are very silly.” I heard this so many times and I think in a way I might be trying to prove, “Look I'm doing a PhD!”* (Woman, 36, UK)

*These things that I have trouble with now, all of it is just minor things. Because in comparison with how it used to be, I’m just happy to be alive*. (Woman, 42, Sweden)

Reflecting on difficult past experiences in education, many people described a persistent ‘inner critic’ that continually questioned their place in the academy; an example of internalised ableism [49]. Participants disclosed the shame they felt when unable to complete tasks in the same way or to the same timescale as peers, with forgetting, lateness, and “*erratic productivity*” cited as particular sources of shame. Competing or overlapping conceptualisations of ADHD — as a psychiatric disorder, disability, and/or neurodivergence — influenced how ‘deserving’ of support they judged themselves to be.

*I recently applied to [funding scheme] and there was a section that said you get a little bit more funding if you have any disability and ADHD was under that section. I think that is my problem. Is it really a disability? Or am I biased because I have ADHD and I just don’t want to accept the fact that it is a disability? Because it is for others. It’s a little bit difficult to understand*. (Woman, 36, UK)

Participants were sensitive to reductive societal stereotypes, such as the ‘fidgety little boy’ trope; misinformation attributing ADHD to bad parenting or lifestyle choices; the idea that people with ADHD are unlikely to be high-achieving or, conversely, the ‘toxic superpower’ narrative [50]. In combination, the experience and expectancy of stigmatised responses from others informed a widely expressed reluctance to “*use ADHD as an excuse*”. This was compounded by the idea that to admit weakness within highly competitive and hierarchical academic workplaces is to self-sabotage. Navigating these complexities made decisions about whether, when, and how to disclose ADHD in the workplace very difficult.

*I didn’t wanna disclose it and I think it’s because I still feel a slight shame about it, even though there was all the support. I didn’t want the examiners to read my thesis and think, “This is a good thesis for someone with autism and ADHD”. I just wanted it to be a good thesis. […] Despite the fact that I know […] the research we provide is different and it is valuable and I have all the academic arguments for it, emotionally I still don’t feel like that*. (Man, 29, Australia)

Internalised stigma related to adverse experiences of education in childhood, in combination with unhelpful societal discourses of ADHD, reduced the likelihood of participants seeking workplace accommodations. Further, the anticipation of being judged as incompetent sometimes disincentivised people from applying for opportunities that might support career progression, an example of stereotype threat [51]. A supportive relationship with a supervisor or mentor who was understanding about ADHD could be protective in minimising the negative impact of stereotype threat. Optimally, supervisors took a flexible approach to research supervision, offered constructive support when things went wrong, and encouraged self-advocacy by early-career researchers in the wider university setting.

*She’s a brilliant academic, but more than that, she’s a very nice person. […] She told me that I need to be a little bit self-compassionate to myself because I’m criticising myself a lot. […] It was a moment that I realised, “OK I am doing a lot of things that, just because I’m able to do it, that doesn’t mean that I should do it.”* (Woman, 36, UK)

Individual functioning of early-career academics with ADHD in the workplace is shaped by the psychological impacts of lifelong experiences of not fitting in and feelings of inadequacy. Many participants in this study described struggling with perfectionism, anxiety, low self-esteem, and internalised stigma, which affected decisions about disclosure, help-seeking, and self-advocacy in the workplace. These challenges to wellbeing were perpetuated in hyper-competitive workplaces, where people perceived that both neurodivergence and mental health challenges would be construed as signs of incompetence.

#### 2c. ADHD and collective academic functioning.

*I think just recognising that ADHD people understand the world differently and that will be inherently beneficial to academia […]. But I don’t necessarily think academia is set up to allow those perspectives to thrive at the moment*. (Man, 29, Australia)

An ecological functions model of neurodiversity would predict advantages of diverse neurocognitive processing styles in the workplace for collective outcomes, which might include scientific progress or workplace cohesion. Despite wide heterogeneity in specialism and career stage, commonly identified research strengths among the participants included idea generation, a proclivity to challenge received wisdom, and an instinctive ability to recognise patterns and integrate diverse perspectives. Methodological pluralism and/or an interdisciplinary approach were common among the interviewees, and generally regarded as an advantage in collaborative research teams.

*I think I definitely make connections better than most […]. Probably this is partly as a consequence of my ADHD, I have such a diverse background in terms of experiences. So I know way more methodologies and theories than a lot of people do. I may not be a master of many of them in the way that somebody who’s done one thing all of their life will be. But I’ll be able to see that bigger picture better than almost anyone else*. (Man, 37, Netherlands)

*I think there is some sort of intuition that comes with it, because maybe you just paid attention to so many things in this peripheral way that at some point it all just comes together*. (Woman, 40 UK)

A highly focused specialism might generally be conducive to research quality and career progression. However, the tendency to make intuitive connections at the ‘big-picture’ level described by these participants could be necessary for a whole field of knowledge to move forward. Simultaneously, it might be either advantageous or restrictive for the individual researcher, depending on context. Other participants described the feeling of “*going down a rabbit-hole”* when driven to find a piece of information or the best technique to address a methodological problem. This hyper-focus might come at the cost of sleep or ‘productivity’ at the individual level but facilitate the progress of a research lab collectively.

*I feel compelled because I have to find the best way that’s going to be the most reliable, that’s going to give me the best results. But then I also feel equally infuriated and frustrated with myself, because every step deeper I go, I know I’m not supposed to be doing it*. (Woman, 25, Australia)

Several participants identified a strong impetus to drive societal change through their research, which was not always incentivised in career progression structures. Again, an individual commitment to translational science and research impact might benefit research groups, while conferring mixed benefits for the individual.

*Part of [my academic identity] might be very action-oriented. As ADHD, I’m like, “OK, I wanna do something.” But also the fact that I know there’s so many research projects where it’s like, “I did this and now it’s going to go into an academic journal and sit there.” I’m like, […] “Why are you doing this if it’s not moving something forward?”* (Woman, 34, Canada)

*Like research has to be ‘sexy’ sometimes or popular, you know. So that’s […] something that’s been quite hard. […] Because for me the most valuable thing is the usefulness but for them, it might not be*. (Woman, 24, Belgium)

Research values and priorities were not uniform across the sample. Many participants identified societal impact as a core motivating value, with academic cultures that incentivised ‘productivity’ in terms of publication rates and grant capture as antithetical to doing meaningful research. However, disconfirming case analysis identified instances in which a competitive workplace was motivational for individuals.

*Since I kind of like to compete, I too easily get into […] what you need to produce and generate publications, which I’m not comfortable with. So I’m struggling with getting those values balanced. […] I don’t value competition. But I kind of like it, I think it’s fun*. (Woman, 42, Sweden)

Across the dataset, interviewees were highly aware of their individual difficulties at work and in other aspects of life. Nonetheless, participants were clear about the strengths that neurodivergent people *can* bring to academic work if workplaces are sufficiently inclusive, including unconventional ways of thinking and problem-solving, creativity, innovation, and a propensity to challenge authority. Given how well these traits serve the goals of academic work, the fact that many universities were experienced as unwelcoming of neurodivergent perspectives was frustrating and even absurd.

*There are so many masking neurodivergent people in academia. […] A friend of mine, who is also ADHD, is working in a lab and they were really scared of disclosing their diagnosis. […] They were trying so hard to fit in and just function in their position. But it turns out ninety percent of their lab is neurodivergent and everyone is just faking it and is miserable, which is so ironic*. (Woman, 30, UK)

Ultimately, individuals with an ADHD neurotype can make distinctive and vital contributions to the collective progress of research groups, departments, institutions, and disciplines. Meanwhile, the capacity of an individual with ADHD to function ‘successfully’ in academia is highly dependent on context.

## Discussion

This study analysed first-person accounts of early-career researchers with ADHD in order to uncover neurotype–environment (mis-)matches that contribute to academic functioning. Informed by an ecological model of neurodiversity [11], we were interested in functioning at both individual and collective levels. Our analysis surfaced examples of fit and friction for ADHDers working in academic environments. Individual traits identified by our participants as conducive to academic work mirror ADHD workplace strengths identified in previous literature, including autoethnographic accounts of academics. These include high trait curiosity [26], inclination to hyperfocus [52] and an ability to recognise holistic patterns [53]. Conversely, individual traits that participants perceived as impeding academic functioning included unpredictable motivation/energy patterns [16], difficulties with organising and planning [54] and task initiation and completion [53].

Crucially, the manifestation of these individual strengths and challenges is context dependent [55]. Several indicators of person-environment fit highlighted in Hotte-Meunier et al.’s systematic review [8] were referenced by participants: a stimulating workplace environment, challenging and novel (yet achievable) work tasks, active learning, and personal autonomy. In the broader employment literature, these ‘fit factors’ mediate the relationship between the strengths and challenges associated with ADHD and employment outcomes, including job satisfaction, tenure, and workplace performance [8].

Notably, however, the relative autonomy and flexibility characteristic of academic work were of mixed benefit to the participants. While the freedom to pursue curiosity-driven research and to work in alignment with energy cycles was a ‘*sweet spot*’, the unbounded nature of academic work meant that early career researchers were often caught in a ‘*juggle-crash cycle*’, in which over-commitment during periods of high energy led to frequent periods of burnout and contributed to other mental health challenges. Where participants were flourishing, they were usually employed in contexts where they could construct their own niche (e.g., build a lab group), work within teams where colleagues compensated for areas of difficulty, and/or have a supervisor who accommodated unpredictable fluctuations in activity and supported self-advocacy within the institution [56].

A critical stance towards a focus on ‘productivity’ over rigorous intellectual inquiry in contemporary academia was frequently (though not ubiquitously) expressed. This stance is common in studies of autistic academics [57] and in broader debates about the purpose of post-secondary education [58]. Alongside a perception of ever-increasing administrative demands and managerialism, this led many participants to experience academia as a non-inclusive environment, or even as actively hostile to neurodivergent ways of working. *Effortfully staying between the lines* meant masking ADHD traits, avoiding disclosure in the workplace and working doubly hard to meet institutional demands. This dynamic was contrasted with institutional initiatives to provide accommodations for neurodivergent students. If universities are meaningfully committed to neuro-inclusion, this means prioritising inclusive practice for all members of the university community [59].

Ecological function models posit that neurodiversity can be advantageous at the group level because it increases the cognitive resources available to adapt to changing environments [11]. Which individual cognitive profiles function best at a given time depends on how societal/ institutional environments are constructed. The participants in the current study worked in a variety of academic disciplines, institutions, and countries. Specific features of their working environment influenced how successfully they perceived that they functioned within their role. However, our analysis identified some common causal tendencies. *Non-traditional career trajectories*, including multiple switches between sectors and patchwork careers combining multiple roles, were typical among participants. This aligns with population-level data showing that the incidence of job switching is elevated among ADHDers [60], likely attributable to a combination of intrinsic characteristics, such as impulsivity and novelty seeking, and non-inclusive working environments. In academia, this propensity can limit career progression in contexts where a coherent track record of programmatic research is prioritised; conversely, it can be advantageous in interdisciplinary research groups or roles that draw on a wider skillset.

A second crucial aspect of individual workplace function related to *maintaining wellbeing.* The high incidence of poor mental health in adults with ADHD is well documented [61] and was reflected in the accounts of our participants. Negative self-concept developed through cumulative experiences of being misunderstood in educational contexts and of other people’s stigmatising responses to ADHD-related behaviours. Stereotype threat often underpinned participants’ reluctance to disclose their diagnosis, “use ADHD as an excuse,” or even to put themselves forward for workplace opportunities [62]. While there were some counter-examples in the data, our analysis suggests that contemporary educational environments (both school and university) are generally not constructed to allow people with an ADHD neurotype to function at their best.

Nonetheless, at the collective level, it is plausible that some of the individual traits and behaviours described by participants are beneficial, even as they cause workplace challenges for individuals. For example, a tendency to generate ideas, make connections between disciplines, and adopt a pluralistic approach to research methods, in virtue of “casting a wide epistemic net” [63, p. 914] may advance knowledge in a way that could not be achieved solely by researchers who stay within strict disciplinary or methodological boundaries. Likewise, a tendency to prioritise inclusive classroom practice and student support might benefit departmental culture, while doing little to advance individual career progression within institutional contexts that chiefly reward research outputs.

### Towards practical recommendations

Given the targeted nature of our sample and the interpretive nature of our analysis, we cannot yet propose concrete recommendations for universities and administrators to support ADHDers in academia. Nonetheless, our findings highlight useful ways to think about relevant issues, informing how future work might develop such recommendations.

First, organisational procedures should bear in mind the tensions and synergies identified above and prioritise thinking holistically about solutions. For example, we described how flexibility is appealing to ADHDers, but also how this can clash with the unbounded nature of academic work, risking ‘*juggle/crash cycles’*. Thus, plans for policy development should not aim at one such dimension (e.g., enhancing flexibility, for instance via “job crafting”) without also considering these tensions (e.g., managing unboundedness). Conversely, we described how novelty is a common drive for ADHDers. Thus, policies aimed at supporting flexible working should also be evaluated in terms of how they support key motivations such as novelty.

Second, mentors and managers should be aware of the dynamic shifts described here. For example, we described how ADHDers may experience periods of energetic productivity followed by periods of burnout. These periods also had implications for ADHDers’ wellbeing and their estimations of their own performance. Plans for inclusive staff development should thus equip mentors and managers to support non-linear energy rhythms, rather than expect continuous output. Similarly, collaborators might consider how their own contributions can complement ADHD working styles, for instance by choosing to support task prioritisation, task initiation, and flexible milestone tracking aimed at project completion. A related challenge is to recognise these dynamic shifts in the first place, given patterns of masking and ‘*effortful staying between the lines’* described above.

Finally, institutional policies (e.g., for hiring or promotion) should be aware of how ADHD career trajectories might look different from typical trajectories. For instance, we described how ADHDers might prefer to explore widely, connecting currently unrelated methods or problems. While these pluralistic, big-picture approaches can benefit science, they are inherently risky for individual researchers. Similarly, the patterns described here (deep dives, side quests or ‘going down rabbit holes’) will not necessarily yield the usual signals of productivity in academia (e.g., number of papers published). Policies that aim to be inclusive should also rethink evaluation in qualitative, not just quantitative terms. Doing so could articulate explicit roles for neurodiversity in meeting calls for “slow science” [64,65].

Overall, aspirations to be more inclusive of ADHDers in academia should incorporate ADHD lived experiences into the policy development process, including via further qualitative research on those experiences.

### Reflexivity, limitations and future directions

The analysis presented here is inevitably influenced by the experiences of some of the authors as neurodivergent academics. Some of us are ‘insider researchers’ in that we are ADHDers who navigate academic careers; some of us are also early-career researchers. We work across different institutional and national contexts.

These insider positionalities influenced research design and analysis. For example, team discussions drawing on our collective experiences informed our choices to interpret “early career” quite broadly; to include self-identified ADHDers; and to allow flexibility in interview modality and questions. The wide window for “early career” means that participants may have differed in the roles and employment conditions they encountered. Regarding the choice to be more inclusive in recruitment, we note that the impact of diagnosis was not one of our research questions, but that self-identified interviewees reported similar experiences (e.g., in terms of workplace challenges, perceptions of own abilities, or reflections on academic career trajectories) as those with formal diagnoses.

Our positionality also informed our earliest set of codes in part, since we often identified with the experiences our participants related. It was therefore particularly important that we recursively questioned and challenged our analysis as it developed, which we did through numerous revisions of the codes in response to repeated, deep engagement with the interview transcripts, ensuring that the analysis did not merely reflect our prior assumptions. The themes presented here reflect the most salient ‘causal tendencies’ that we arrived at through a lengthy process of analysis, re-analysis and member checking, but other interpretations of the data are equally possible. As an in-depth qualitative study, we did not aim to produce generalisable findings, but we hope the thematic structure presented is transferable to other contexts. In this regard, it is important to note that our sample was predominantly White, based at universities in the Global North, and mostly specialised in psychological and social sciences or cognate disciplines. Our recruitment strategy was necessarily limited to those with sufficient English ability to discuss these complex subjects, but may additionally (and inadvertently) have selected for various features that influenced our interpretive themes, including openness to discuss topics such as diagnosis and disability; familiarity with the neurodiversity paradigm; and adult (as opposed to childhood) diagnosis. Taken together, these features likely inform participants’ experiences of ADHD (including medical/support pathways) and professional life. Similarly, recruitment through our existing professional networks and social media platforms made it easier to reach those in the Global North. Thus, our findings might not transfer to other contexts, including areas where ADHD is more stigmatised, or where professional support for disabilities is more informed by biomedical/deficit paradigms. A more diverse sample may have elicited different findings.

Nonetheless, our study contributes to the small but growing research literature on neurodivergence in academia. To examine the contribution of cognitive diversity in knowledge production contexts more fully, different methodological approaches would be needed that harness larger-scale institutional and national data. This includes testing various contexts (institutions, academic fields, national cultures, etc.) to evaluate *whether* (and if so, *when*) ADHDers’ potential contributions to collective academic progress—currently, reflected in participants’ subjective perspectives and our theoretical interpretations thereof—actually materialise. However, the personal accounts analysed in the current study provide a valuable starting point. It is often speculated that historical figures who led paradigm-shifting scientific discoveries or significant innovations in arts and culture displayed neurodivergent traits. To ensure that unconventional thinkers are still empowered to contribute to knowledge (benefitting collective function), it is critical that we understand how academic environments can inhibit individual function, and that we challenge exclusionary practices and structures in universities.

## Supporting information

S1 File COREQ (COnsolidated criteria for REporting Qualitative research). (DOCX)
