# Supplementary material for: “The significance of context”: Fit, friction, and function in the work lives of early career academics with ADHD
Source: PLoS One. 2026 Oct 29;21(10):e0361208. doi: 10.1371/journal.pone.0361208 (PMC13718954; doi:10.1371/journal.pone.0361208)
Supplement: Supplementary file 1 [file pone.0361208.s001.docx]

**“The significance of context”: Fit, friction, and function in the work lives of early career academics with ADHD**

Supplemental Material 1 (SM1)

COREQ (COnsolidated criteria for REporting Qualitative research)

An anonymous reviewer has requested a COREQ checklist ([Tong et al., 2007](https://doi.org/10.1093/intqhc/mzm042)) as a way to provide further detail on methods and analysis. Here, we provide an adapted version of some of the relevant information, in paragraph format rather than the usual table/checklist format. This allows us to unpack our methodological and analytic decisions in more detail: we care more about *why* things were done than just reporting *what* was done. This adaptation also means that we can reserve some requested information for this supplementary material, rather than reporting all of it in the main text.

Further, we note that Braun and Clarke ([2024](https://doi.org/10.1016/j.metip.2024.100155)) argue that the COREQ is only apt for specific kinds of qualitative analyses, not least because it was developed to meet the specific epistemic needs of certain streams within health research. Our analytic approach aligns more with the critical approach of Braun and Clarke, which renders some of the more positivist-aligned COREQ items less relevant.

# Domain 1: Research team and reflexivity

## Personal Characteristics

**1. Interviewer/facilitator:** **Which author/s conducted the interview or focus group?**

AR, LH, JS

Not listed on a specific page of the manuscript. It’s only mentioned that four members of the research team conducted the interviews. One interviewer left the project team before we began drafting the manuscript, and that person chose not to be listed as an author. Here (and in response to subsequent items), we only provide information on the personal characteristics of the three interviewers listed as authors.

**2. Credentials:** **What were the interviewers' credentials?**

AR: BS (at the time of interviewing) and, subsequently, MS

LH: PhD
JS: PhD

Not listed on a specific page of the manuscript.

**3. Occupation:** **What was their occupation at the time of the study?**
AR: Student (at the time of interviewing) and, subsequently, Research Assistant

LH: Professor of Developmental Psychology and Inclusive Education
JS: Postdoctoral researcher

Not listed on a specific page of the manuscript.

**4. Gender:** **What was the researcher’s gender?**

AR: Woman

LH: Woman
JS: Man

Not listed on a specific page of the manuscript.

**5. Experience and training: What experience or training did the researcher have?**

AR: Prior experience conducting qualitative research through bachelor’s and master’s research projects; took a course on qualitative research during bachelor’s degree; experience with qualitative interviewing and analysis.

LH: Extensive experience conducting qualitative research (interviewing and analysis); took the lead on the qualitative analysis in this paper.
JS: Training during the initial stages of the project, with mock interviews and collective reflection on performance, including feedback from experienced qualitative researchers.

Not listed on a specific page of the manuscript.

## Relationship with participants

**6. Relationship established:** **Was a relationship established prior to study commencement?**

AR: No

LH: No
JS: No

Not listed on a specific page of the manuscript.

**7. Participant knowledge of interviewer:** **What did participants know about the researcher?**

As mentioned in the manuscript, some of the researchers on the authorship team are insider researchers: they have experience navigating academic careers and/or have ADHD. Depending on whether the interviewer had lived experience of ADHD and/or had worked in academia, they may or may not have disclosed this to the participants they interviewed to build rapport or as a reason for doing the research. As we are not disclosing which member(s) of the research team is/are neurodivergent, we cannot be more specific than that.

Not listed on a specific page of the manuscript.

**8. Interviewer characteristics:** **What characteristics were reported about the interviewer/facilitator?**

See response to Item 7.

Not listed on a specific page of the manuscript.

# Domain 2: Study design

## Theoretical framework

**9. Methodological orientation and Theory: What methodological orientation was stated to underpin the study?**

The study took a critical realist approach to thematic analysis ([Fryer, 2022](https://doi.org/10.1080/14767430.2022.2076776); [Wiltshire & Ronkainen, 2021](https://doi.org/10.1080/14767430.2021.1894909)). This is a qualitative analytic framework partly rooted in reflexive thematic analysis ([Braun & Clarke, 2006](https://doi.org/10.1191/1478088706qp063oa); [2022](https://doi.org/10.1037/qup0000196)).

Mentioned on page 15 under the heading "Data analysis".

## Participant Selection

**10. Sampling:** **How were participants selected?**

Recruitment combined features of purposive sampling (by design, we targeted early-career academics with ADHD) and convenience sampling (we recruited by advertising in our established professional networks and on social media)

Mentioned on page 11 under the heading "Participants".

**11. Method of approach: How were participants approached?**

Potential participants approached the research team via email, via the Slack channel of a group of neurodivergent academics, and via social media. Recruitment materials advised interested participants to email the research team. We did not approach the prospective participants directly, except for one participant (contacted via email): this participant was indirectly known to the research team via an online network.

Briefly summarised on page 11.

**12. Sample size:** **How many participants were in the study?**

Twenty interviews were conducted. As noted in the main text, "sample size justification" is an ill-defined term in qualitative research. Nonetheless, our plan to recruit 20 interviewees is consistent with recent guidance ([Wutich et al., 2024](https://doi.org/10.1177/16094069241296206)) and with guidelines offered by Braun and Clarke (<https://www.thematicanalysis.net/faqs/>) for a medium-sized project.

Listed on page 12 of the manuscript.

**13. Non-participation: How many people refused to participate or dropped out?**

Ten prospective participants who initially expressed interest via email stopped responding to follow-up emails from the team to confirm their continued interest in taking part. Three prospective participants expressed interest but did not meet all eligibility criteria. One prospective participant declined to participate after initially expressing interest, citing difficulty finding a mutually convenient time with an interviewer on the team, following several back-and-forth emails. One prospective participant had to cancel the interview on the day of the interview due to poor internet connectivity.

Not listed on a specific page of the manuscript.

## Setting

**14. Setting of data collection: Where was the data collected?**

For the pre-interview survey of language preferences and demographic questions, data were collected online via Qualtrics. Interviews were conducted online via video-conferencing platforms such as Microsoft Teams or Zoom.

The use of Qualtrics is not reported on any page in the manuscript, but the use of video-conferencing platforms is reported on page 14 of the manuscript.

**15. Presence of non-participants: Was anyone else present besides the participants and researchers?**

No.

Not listed on a specific page of the manuscript.

**16. Description of sample: What are the important characteristics of the sample?**

Interviewees included 11 women, 8 men, and one non-binary person. They were from 10 different countries, all categorised as part of the Global North. The UK had the most participants (5), followed by the US and Canada, with 3 participants each. Participants were predominantly White (85%), and the rest (15%) were people of color, including one individual with a multi-racial background. 85% of participants had an official diagnosis of ADHD, and 65% had co-occurring mental health conditions or were neurodivergent in other ways. The participants were employed in academia: half were doctoral students, while almost all other participants were lecturers/assistant professors or in postdoctoral roles. The majority (55%) reported that Psychology was their subject specialisation.

Demographics are detailed on pages 12-13 of the manuscript.

## Data collection

**17. Interview guide: Were questions, prompts, guides provided by the authors? Was it pilot tested?**

Prior to interviews, participants were informed that they could request a copy of the semi-structured interview guides in advance, if they desired.

The interview procedure was pilot-tested twice: mock interviews were conducted with colleagues of the research team who met the inclusion criteria (but whose interviews were not included in the final dataset for analysis).

These details are included on page 14 of the manuscript under “Data collection”.

**18. Repeat interviews:** **Were repeat interviews carried out? If yes, how many?**
No repeat interviews were carried out, though one interview was divided into two sessions, as the interviewer felt unwell during the first part of the interview and needed to reschedule.

Not listed on a specific page of the manuscript.

**19. Audio/visual recording:** **Did the research use audio or visual recording to collect the data?**

Yes. Interviews were carried out over the video conferencing platforms Zoom and Microsoft Teams, which have built-in audio-visual recording features. All interviews were audio and video recorded with the consent of participants.

This information is mentioned on page 14 of the manuscript.

**20. Field notes:** **Were field notes made during and/or after the interview or focus group?**

Interviewers took brief field notes during the interviews, in order to:

- Capture potential directions for follow-up questions (e.g., potential clarification questions or points of ambiguity);
- Highlight salient points of interest, including possible connections with other parts of the interview (or other interviews), as well as striking phrases or word choices;
- Summarise key points while fresh in memory, aiding later transcription;
- Note non-verbal cues where relevant;

Not listed on a specific page of the manuscript.

**21. Duration:** **What was the duration of the interviews or focus group?**

Interviews ranged between 41 and 110 minutes, with an average duration of 65.95 minutes (SD = 17.61).

This information is on page 14 of the manuscript.

**22. Data saturation:** **Was data saturation discussed?**

Data saturation was not discussed as it is not relevant to this kind of analysis ([Braun & Clarke, 2024](https://doi.org/10.1016/j.metip.2024.100155)). Saturation is often mentioned in relation to sample size. We used guidelines for sample size suggested by Braun and Clarke (<https://www.thematicanalysis.net/faqs/>) for a medium-sized project, while noting that those same authors stress that "sample size" is an ill-defined concept for qualitative analyses of this type. Our initial target sample size was between 15 and 30 participants.

Not listed on a specific page of the manuscript.

**23. Transcripts returned:** **Were transcripts returned to participants for comment and/or correction?**

No. Interviews were transcribed verbatim based on audiovisual recordings. Recordings were of sufficiently high quality that the transcription process was relatively straightforward overall. However, even if participants did not comment on the entire transcripts, they were able to comment on the quotations that appeared in the draft manuscript prior to submission. See Item 28 (Participant checking) for more information.

Not listed on a specific page of the manuscript.

# Domain 3: analysis and findings

## Data Analysis

**24. Number of data coders: How many data coders coded the data?**

Two authors coded the data.

Listed on page 16 of the manuscript.

**25. Description of the coding tree: Did authors provide a description of the coding tree?**

Our analytic approach does not involve a coding tree. However, to provide an analytic trail, the project's Open Science Framework repository (<https://osf.io/hb32d/>) provides examples of initial codes, illustrates potential groupings of those codes, and shows how updated codes were related to the eventually constructed themes.

The process of creating the coding scheme is briefly described on pages 16-17 of the manuscript.

**26. Derivation of themes: Were themes identified in advance or derived from the data?**

Themes were derived from the data, based on iterative rounds of inductive coding and abductive reasoning (integrating relevant theoretical knowledge to construct explanatory themes).

The process of theme development is described on pages 16-17 of the manuscript, with concrete examples provided in the projects Open Science Framework repository (<https://osf.io/hb32d/>).

**27. Software: What software, if applicable, was used to manage the data?**

Initially, Author 1 used paper, pencil, and printouts of the transcripts to note initial codes, and then subsequently transitioned the codes to Microsoft Excel. Author 2 used NVivo for the initial round of coding. Following discussion and integration of both authors’ coding schemes, Microsoft Excel was used to reduce and refine the coding scheme, as detailed in the analytic trail available via the project's Open Science Framework repository (<https://osf.io/hb32d/>)

Software programs are not mentioned on any page of the manuscript.

**28. Participant checking:** **Did participants provide feedback on the findings?**

Yes, we received feedback from seven participants, after all participants were sent a link to the draft manuscript before submission.

Participant feedback was overwhelmingly positive, with respondents noting that the findings deeply resonated with their lived experiences, for instance, describing a sense of validation and recognition regarding “patchwork” careers, hyperfocus, and the emotional toll of academic structures.

There were two participant comments in particular that led to minor corrections to the manuscript:

1. One participant's feedback highlighted how a paragraph about marking deadlines missed an important piece of context: "I did also see my quotes in there which was pleasing... I just wondered whether the paragraph before my quote on Page X might be clearer if it was more explicit as the subject was the 5-day extension for students with ADHD, but it wasn't clear that this was the student's benefit. I think the context is a little out as I was saying that if I couldn't meet the marking deadline because I was having difficulty marking because of my ADHD, that my manager would give the part I couldn't finish to another lecturer…"

Accordingly, we amended this section to highlight the disparity between formalised extensions for students versus the informal, guilt-laden redistribution of uncompleted work among colleagues for academic staff.

2. Another detailed piece of feedback highlighted how burnout is frequently driven by systemic workloads as opposed to purely individual limitations: "I do think that your current description seems to focus a bit too much on the individual factors behind it ('periods of high energy led to frequent periods of burnout'), but I think it is structural factors that lead to the creation of that cycle... You spend so much time thinking 'I can't do the work because I am bad at it' that you don't realise that 'I am great at the job, but the quantity is so high that NO ONE could do all the work' is even a possibility."

We integrated this perspective into our analysis of the burnout cycle to ensure that the manuscript captures both individual experiences and systemic contexts.

Member checking is described on page 11 of the manuscript.

## Reporting

**29. Quotations presented: Were participant quotations presented to illustrate the themes/findings? Was each quotation identified?**

Participant quotations were used to illustrate the themes. Quotations were identified with information about the participant demographics: i.e., gender, age, and country.

Quotations are included throughout the Results section of the manuscript.

**30. Data and findings consistent: Was there consistency between the data presented and the findings?**

Yes, we believe so. However, we note Braun and Clarke's ([2024](https://doi.org/10.1016/j.metip.2024.100155)) critique of COREQ's assumption of a positivist epistemology. Our findings are not simply "facts" that can be excavated from the presented quotations (the "data"), but are inherently interpretive. Hence, we leave the assessment of this item to the reviewers and readers.

**31. Clarity of major themes: Were major themes clearly presented in the findings?**

The themes are made explicit in the structure of subheadings in the Results section, and in the thematic map presented in Figure 1.

**32. Clarity of minor themes: Is there a description of diverse cases or discussion of minor themes?**

Relevant facets of both main themes are made explicit in the structure of subheadings under Results, and in Figure 1.

Disconfirming case analysis is described in the Method (p. 10, p. 17) with a specific example highlighted in the Results (p. 33).
