# Supplementary material for: “The significance of context”: Fit, friction, and function in the work lives of early career academics with ADHD
Source: PLoS One. 2026 Oct 29;21(10):e0361208. doi: 10.1371/journal.pone.0361208 (PMC13718954; doi:10.1371/journal.pone.0361208)
Supplement: Peer review history

# Peer review: Decision Letter 0

**DOI:** 10.1371/journal.pone.0361208.r001

**Authors:** Branko Aleksic

6 Jul 2026

Dear Dr. Hamilton,

Thank you for submitting your manuscript to PLOS ONE. After careful consideration, we feel that it has merit but does not fully meet PLOS ONE’s publication criteria as it currently stands. Therefore, we invite you to submit a revised version of the manuscript that addresses the points raised during the review process.

plosone@plos.org. When you're ready to submit your revision, log on to https://www.editorialmanager.com/pone/ and select the 'Submissions Needing Revision' folder to locate your manuscript file.

- A letter that responds to each point raised by the academic editor and reviewer(s). You should upload this letter as a separate file labeled 'Response to Reviewers'.
- A marked-up copy of your manuscript that highlights changes made to the original version. You should upload this as a separate file labeled 'Revised Manuscript with Track Changes'.
- An unmarked version of your revised paper without tracked changes. You should upload this as a separate file labeled 'Manuscript'.

If you would like to make changes to your financial disclosure, please include your updated statement in your cover letter. Guidelines for resubmitting your figure files are available below the reviewer comments at the end of this letter.

If applicable, we recommend that you deposit your laboratory protocols in protocols.io to enhance the reproducibility of your results. Protocols.io assigns your protocol its own identifier (DOI) so that it can be cited independently in the future. For instructions see: https://journals.plos.org/plosone/s/submission-guidelines#loc-laboratory-protocols. Additionally, PLOS ONE offers an option for publishing peer-reviewed Lab Protocol articles, which describe protocols hosted on protocols.io. Read more information on sharing protocols at https://plos.org/protocols?utm_medium=editorial-email&utm_source=authorletters&utm_campaign=protocols.

As the corresponding author, your ORCID iD is verified in the submission system and will appear in the published article. PLOS supports the use of ORCID, and we encourage all coauthors to register for an ORCID iD and use it as well. Please encourage your coauthors to verify their ORCID iD within the submission system before final acceptance, as unverified ORCID iDs will not appear in the published article. *Only*  the individual author can complete the verification step; PLOS staff *cannot*  verify ORCID iDs on behalf of authors.

We look forward to receiving your revised manuscript.

Kind regards,

Branko Aleksic, MD, PhD

Academic Editor

PLOS One

Journal Requirements:

When submitting your revision, we need you to address these additional requirements.

1.Please ensure that your manuscript meets PLOS ONE's style requirements, including those for file naming. The PLOS ONE style templates can be found at

https://journals.plos.org/plosone/s/file?id=wjVg/PLOSOne_formatting_sample_main_body.pdf and

https://journals.plos.org/plosone/s/file?id=ba62/PLOSOne_formatting_sample_title_authors_affiliations.pdf

2. We note that this data set consists of interview transcripts. Can you please confirm that all participants gave consent for interview transcript to be published?

If they DID provide consent for these transcripts to be published, please also confirm that the transcripts do not contain any potentially identifying information (or let us know if the participants consented to having their personal details published and made publicly available). We consider the following details to be identifying information:

- Names, nicknames, and initials

- Age more specific than round numbers

- GPS coordinates, physical addresses, IP addresses, email addresses

- Information in small sample sizes (e.g. 40 students from X class in X year at X university)

- Specific dates (e.g. visit dates, interview dates)

- ID numbers

Or, if the participants DID NOT provide consent for these transcripts to be published:

- Provide a de-identified version of the data or excerpts of interview responses

- Provide information regarding how these transcripts can be accessed by researchers who meet the criteria for access to confidential data, including:

a) the grounds for restriction

b) the name of the ethics committee, Institutional Review Board, or third-party organization that is imposing sharing restrictions on the data

c) a non-author, institutional point of contact that is able to field data access queries, in the interest of maintaining long-term data accessibility.

d) Any relevant data set names, URLs, DOIs, etc. that an independent researcher would need in order to request your minimal data set.

For further information on sharing data that contains sensitive participant information, please see: https://journals.plos.org/plosone/s/data-availability#loc-human-research-participant-data-and-other-sensitive-data

If there are ethical, legal, or third-party restrictions upon your dataset, you must provide all of the following details (https://journals.plos.org/plosone/s/data-availability#loc-acceptable-data-access-restrictions):

1. A complete description of the dataset

2. The nature of the restrictions upon the data (ethical, legal, or owned by a third party) and the reasoning behind them

3. The full name of the body imposing the restrictions upon your dataset (ethics committee, institution, data access committee, etc)

4. If the data are owned by a third party, confirmation of whether the authors received any special privileges in accessing the data that other researchers would not have

5. Direct, non-author contact information (preferably email) for the body imposing the restrictions upon the data, to which data access requests can be sent

3. We notice that your supplementary figures are uploaded with the file type 'Figure'. Please amend the file type to 'Supporting Information'. Please ensure that each Supporting Information file has a legend listed in the manuscript after the references list.

4. Please include your full ethics statement in the ‘Methods’ section of your manuscript file. In your statement, please include the full name of the IRB or ethics committee who approved or waived your study, as well as whether or not you obtained informed written or verbal consent. If consent was waived for your study, please include this information in your statement as well.

5. If the reviewer comments include a recommendation to cite specific previously published works, please review and evaluate these publications to determine whether they are relevant and should be cited. There is no requirement to cite these works unless the editor has indicated otherwise.

Additional Editor Comments:

Thank you for choosing our journal for your work. See the comments below.

[Note: HTML markup is below. Please do not edit.]

Reviewer's Responses to Questions

**Comments to the Author**

1. Is the manuscript technically sound, and do the data support the conclusions?

Reviewer #1: Yes

Reviewer #2: Yes

**********

2. Has the statistical analysis been performed appropriately and rigorously?

Reviewer #1: N/A

Reviewer #2: N/A

**********

3. Have the authors made all data underlying the findings in their manuscript fully available?

The PLOS Data policy

Reviewer #1: Yes

Reviewer #2: Yes

**********

4. Is the manuscript presented in an intelligible fashion and written in standard English?

Reviewer #1: Yes

Reviewer #2: Yes

**********

Reviewer #1: Recommendation

Major revision

Summary

This manuscript reports a qualitative study of the work lives of early-career academics with ADHD. Drawing on semi-structured interviews with 20 participants across 10 countries, the authors use a critical realist thematic analysis to examine neurotype-environment fit and friction in academic workplaces, and to consider how ADHD may matter for both individual career trajectories and collective knowledge production.

I found the manuscript engaging, timely, and potentially valuable. The topic is under-studied, the first-person accounts are rich, and the ecological framing provides a useful way to move beyond a simple deficit-versus-strengths account of ADHD. However, several points require clarification before publication, especially the strength of the collective-function claims, the transparency of the analytic process, and the implications of the sample composition.

Major comments

1. Please moderate and clarify the claims about collective academic functioning.

One of the manuscript's most interesting contributions is the argument that ADHD-related traits may benefit collective academic functioning, for example through idea generation, interdisciplinary thinking, pattern recognition, or challenging established assumptions. This is a valuable and original angle. However, the empirical basis for this claim appears to be mainly participants' own reflections on their work styles and perceived contributions. That is appropriate for a qualitative study of first-person experience, but it does not directly demonstrate collective-level outcomes in research groups, departments, or disciplines.

I recommend distinguishing more explicitly between participants' perceptions of their contributions, the authors' theoretical interpretation of those perceptions through an ecological model, and any stronger claim about actual benefits to collective knowledge production. The manuscript would be more convincing if the collective-function argument were framed as theoretically plausible and grounded in participants' accounts, rather than as an empirically demonstrated outcome of the study.

2. Please provide more detail on the analytic process and reflexivity.

The methods section describes the use of critical realist thematic analysis and refers to Fryer's five analytic steps. This is helpful, but the reader would benefit from more concrete detail about how the analysis moved from transcripts to codes, subthemes, and the two final themes. For example, it would be useful to clarify how initial codes were generated, how coding differences were discussed, how theoretical concepts were introduced into the coding scheme, and how disconfirming cases affected the final thematic structure.

Relatedly, the authors appropriately disclose that some members of the team are neurodivergent academics and describe reflexive practices such as journaling, team discussion, disconfirming-case analysis, and member checking. This is a strength. The paper would be stronger if it gave a brief example of how insider positionality shaped recruitment, interviewing, interpretation, or theme development. More detail on member checking would also help: what materials participants received, what feedback was provided, and what kinds of amendments were incorporated.

3. Please discuss the sample composition and recruitment strategy more directly.

The sample is appropriate for an exploratory qualitative study, but it is also quite specific. Participants were recruited through online academic and neurodiversity networks, were willing to discuss ADHD in an interview, were sufficiently fluent in English, and were mostly White, based in the Global North, and concentrated in psychology, education, neuroscience, biological sciences, and social sciences. These features likely shaped the themes. For example, participants connected to neurodiversity networks may be more likely to use neurodiversity-affirming language or to interpret their experiences through that framework.

The limitations section notes some of these points, but the manuscript could go further in explaining how they may have influenced the findings. The paper should avoid implying that the themes necessarily describe early-career academics with ADHD more broadly. The findings may be better framed as a detailed account of a particular, theoretically informative group of participants.

4. Please clarify how diagnostic status was handled analytically.

The decision to include both formal ADHD diagnosis and self-identification is understandable, especially given barriers to ADHD assessment. The manuscript states that 17 of 20 participants had a clinical diagnosis, but it is not clear whether or how diagnostic status was considered during analysis. Were the three self-identified participants' accounts broadly similar to those of diagnosed participants? Did any themes depend on diagnosis-related experiences, such as access to accommodations, disclosure, or reframing past educational experiences?

The authors do not need to separate the groups formally, and doing so may not be useful with this sample size. However, a short explanation of how diagnostic status was treated analytically would increase confidence in the interpretation.

Minor comments

1. The abstract is informative but dense. Consider simplifying the description of the ecological contribution so that the central research question and main findings are easier to grasp for non-specialist readers.

2. The terms "function", "functioning", "collective functioning", and "academic functioning" are central to the manuscript. A concise definition early in the paper would help readers follow the argument.

3. The discussion would benefit from a short, concrete statement of practical implications for universities, such as accessible administrative processes, supervisor training, workload management, or accommodation processes co-designed with neurodivergent academics.

4. The paper defines early-career academics as including PhD students through 10 years post-PhD. Because this is a broad span with different institutional power and employment conditions, the authors could acknowledge that "early career" may encompass quite different workplace experiences.

Confidential comments to the editor

I recommend major revision. The manuscript addresses an important and under-researched topic and is likely to interest PLOS ONE readers. The qualitative data are rich, and the ecological framing is promising. My main concerns are not about the value of the study, but about the precision of the claims made from the data and the need for more methodological transparency. I would be supportive of publication if these issues are addressed.

Reviewer #2: Dear authors,

It is a pleasure to review a clearly written manuscript on such an interesting and important topic. The manuscript has several strengths, including an explicit discussion of reflexivity, a clearly stated critical realist approach, and a careful, well-designed interview process that takes participants’ language and communication preferences into account. However, some points would benefit from further clarification and justification. As noted below, I suggest consulting the COREQ and/or SRQR reporting guidelines and, if appropriate, including a completed checklist as supplementary material to improve the methodological reporting of the manuscript.

Major suggestions

1. Strengthen COREQ-aligned reporting

Several important COREQ items are missing or only partially reported. It would be useful to explicitly state which authors conducted the interviews; the interviewers’ credentials, occupations, gender, and relevant experience or training; whether any prior relationship existed with participants; what participants knew about the interviewers’ positionality or reasons for conducting the study; whether non-participants were present; whether repeat interviews occurred; whether field notes were taken; whether transcripts were returned to participants; what software, if any, was used; and whether there were any cases of non-participation, refusal, ineligibility, or dropout. A supplementary COREQ table would address this efficiently.

2. Clarify the conceptual status of ADHD in the manuscript

From a conceptual perspective, the manuscript would benefit from a clearer account of how ADHD is framed by the authors. The different ways in which ADHD is referred to may create confusion regarding whether it is considered a form of psychopathology, a neurodevelopmental condition, a disability, a non-pathologising identity category, or some combination of these frameworks. This is understandable given the topic, but the authors’ conceptual position should be clearer. This is especially important because the analysis makes claims about “ADHD neurotype” and “causal tendencies.” I suggest adding a short paragraph explaining how the authors understand ADHD in relation to diagnostic criteria, self-identification, neurodiversity-informed language, and psychopathology/disability frameworks.

3. Justify the inclusion of self-identified ADHD participants more explicitly

The manuscript states that formal diagnosis and self-identification were equally acceptable because of barriers to clinical ADHD assessment. This is defensible, but it requires further methodological justification. This point also relates to the previous comment and could be clarified in the same section.

4. Clarify the sampling strategy and sample adequacy

The inclusion criteria are partly described, but the sampling strategy should be named more explicitly, whether purposive sampling, convenience sampling, snowball sampling, or another strategy. The authors should also explain how the final sample size was judged adequate. The current manuscript does not make clear why 20 interviews were sufficient for the analytic claims being made.

5. Make the critical realist analytic process more transparent

The use of critical realist thematic analysis is a strength, but some key analytic terms remain abstract. The manuscript would benefit from a clearer explanation of how the authors moved from participant accounts to codes, then to events, causal tendencies, and explanatory themes. Terms such as “retroductive reasoning” and “causal tendencies” should be briefly operationalised for the reader. A short analytic example, coding tree, or supplementary analytic trail would strengthen the credibility of the analysis.

6. Moderate or further support claims about collective academic functioning

The findings concerning individual experiences, institutional barriers, masking, overcommitment, burnout, and career trajectories are well supported by participant quotations. The claims about collective-level benefits to knowledge production are plausible and theoretically interesting, but they are more inferential. The authors should more clearly distinguish between participant-reported experience, critical realist interpretation, and theoretical extrapolation. Some statements about ADHD academics benefiting knowledge production could be phrased more cautiously unless additional analytic support is provided.

7. Expand the limitations and transferability discussion

The authors appropriately state that the study does not aim for generalisability, but the transferability discussion should be more specific. In addition, the mean age of diagnosis suggests that the sample largely reflects late-diagnosed adults, which may shape retrospective self-understanding and workplace narratives. These issues can be discussed as limitations to transferability.

Minor comments

1. Member checking should be described in slightly more detail.

The use of member checking is a strength. Please clarify how many participants were invited, how feedback was collected, and whether any feedback challenged or changed the thematic structure.

2. Consider moving or expanding some reflexivity material in the Methods.

The reflexivity section is useful, but more COREQ-relevant information should appear in the Methods or supplementary material, particularly interviewer characteristics and researcher–participant relationships.

3. Provide the interview guide or sample questions.

The manuscript describes the interview schedule, but including the full guide or sample questions as supplementary material would improve transparency.

4. Clarify the role of autoethnographic literature in the Discussion.

When comparing the findings with autoethnographic accounts, the authors should clarify that these sources are being used as contextual or interpretive literature, rather than as direct corroborative evidence equivalent to empirical workplace studies.

Overall, this is a promising and carefully conducted qualitative study. Addressing the points above would substantially strengthen the manuscript’s credibility, alignment with qualitative reporting standards, and interpretive clarity.

**********

what does this mean?). If published, this will include your full peer review and any attached files.

If you choose “no”, your identity will remain anonymous but your review may still be made public.

**Do you want your identity to be public for this peer review?**  For information about this choice, including consent withdrawal, please see our Privacy Policy

Reviewer #1: No

Reviewer #2: No

**********

[NOTE: If reviewer comments were submitted as an attachment file, they will be attached to this email and accessible via the submission site. Please log into your account, locate the manuscript record, and check for the action link "View Attachments". If this link does not appear, there are no attachment files.]

To ensure your figures meet our technical requirements, please review our figure guidelines: https://journals.plos.org/plosone/s/figures

You may also use PLOS’s free figure tool, NAAS, to help you prepare publication quality figures: https://journals.plos.org/plosone/s/figures#loc-tools-for-figure-preparation.

NAAS will assess whether your figures meet our technical requirements by comparing each figure against our figure specifications.

# Author comment: Author response to Decision Letter 1

**DOI:** 10.1371/journal.pone.0361208.r002

6 Oct 2026

Response to reviewers (please see formatted version in attached file)

Editor

We note that this data set consists of interview transcripts. Can you please confirm that all participants gave consent for interview transcript to be published?

If they DID provide consent for these transcripts to be published, please also confirm that the transcripts do not contain any potentially identifying information (or let us know if the participants consented to having their personal details published and made publicly available).

Or, if the participants DID NOT provide consent for these transcripts to be published:

- Provide a de-identified version of the data or excerpts of interview responses

- Provide information regarding how these transcripts can be accessed by researchers who meet the criteria for access to confidential data

We understand and appreciate the importance of open data. Open-science values informed our study design and consent procedures from the very start of the project. In line with our ethics boards' decisions, prior to participation, interviewees consented to allowing redacted versions of the transcripts to be made publicly available, with the stipulation that each participant would first be shown and need to approve the redactions for their own interviews.

However, while attempting to carry out the relevant redactions, the research team realised that ethical, anonymous redaction would not be possible. Participants were thus not provided with redacted versions for their approval, so we do not have consent to openly share interview transcripts.

The interviews discussed personal experiences in academia, including research topics, career trajectories, and geographic locations. Even if some of this information could be straightforwardly redacted (e.g., isolated mention of geographic locations, including institutions), the remaining personal topics were holistically and inextricably woven throughout the interview discussions. In our collective judgment, these narrative threads could not be redacted without seriously compromising the value of the interview transcripts as open data.

More concretely, the issue is that the ethical risk of revealing these personal details is not a matter of personal-identifying information contained in isolated utterances. Rather, it is the overall pattern of data that would plausibly help readers in relevant academic communities make inferences about participants' identities. It is this "overall pattern" that we see as a barrier to redaction—but an ethical barrier if not redacted. We think it important to err on the side of caution here for several reasons.

First, we note that the relevant identities (ADHDers in academia) are themselves sensitive information. We cannot assume that all participants are "out" as neurodivergent in their workplaces, and even when participants explicitly mentioned disclosure during their interviews (e.g., to colleagues or supervisors), we cannot assume that those participants are still working at those same institutions, especially given the early-career nature of the sample. We think this situation thus merits utmost caution.

Second, several participants were recruited from our established academic networks. Even relatively few, indirect cues across a transcript might be enough for other readers in those networks to make guesses about participants' identities. As the interviews necessarily focused on personal experiences in academia, very little remaining information would remain in open data if we redacted all such cues. We think the academic value of probing deeply into personal experiences for this study (in the context of our reported themes with carefully curated supporting quotations) far outweighs the knock-on effect to open-science of the transcripts being unredactable.

Third, we note that a similar community—the autistic community—has raised ethical concerns about sharing open data of their personal experiences (https://doi.org/10.1371/journal.pone.0338569). In addition to concerns about identification raised above, these include concerns about misuse of data or potentially harmful narratives arising from secondary researchers' interpretations of their personal experiences. We think our concerns align with guidance regarding qualitative data from vulnerable populations (https://doi.org/10.1111/bjso.12576).

We have updated the manuscript to emphasise these issues (p. 14):

Interview transcripts cannot be made openly available as this is a qualitative study drawing on data that (a) covers sensitive subject matter; (b) carries a high risk of identification of participants due to the specific nature of the sample (early-career researchers with ADHD); (c) by design, reflects personal experiences throughout.

Nonetheless, in order to provide some open data as requested (other than interview transcripts), we have added several data files to the project's repository at the Open Science Framework (https://osf.io/hb32d). These files contain the codes developed during analysis, and trace the evolution of these codes over several iterations of analysis, ultimately showing how they were grouped into themes. This significantly expands on the range of data in the manuscript itself, and provides further information about the analytic process, particularly from an earlier stage of analysis (relative to the ultimate themes reported in the main text).

We notice that your supplementary figures are uploaded with the file type 'Figure'. Please amend the file type to 'Supporting Information'. Please ensure that each Supporting Information file has a legend listed in the manuscript after the references list.

The file uploaded as file type ‘Figure’ is not supplementary, but Figure 1 from the main text. Please see p. 17 of manuscript where location of this figure is identified, along with the following caption:

Figure 1

Thematic map of neurotype-environment interactions and academic functioning. The interplay between neurotype-environment fit and friction (left) and ADHD and academic functioning (right), highlighting the relationship between individual experiences (e.g., the juggle-crash cycle) and broader academic trajectories.

Please include your full ethics statement in the ‘Methods’ section of your manuscript file. In your statement, please include the full name of the IRB or ethics committee who approved or waived your study, as well as whether or not you obtained informed written or verbal consent. If consent was waived for your study, please include this information in your statement as well.

While this information was included in the original manuscript, we have now combined it within a separate subsection, titled ‘Ethics Statement’ (p. 11).

Ethics statement

Primary ethical approval for the study was obtained from the School of Education, Language and Psychology Ethics Committee at York St John University (ETH2223-0023). Participants provided fully informed written consent before taking part in interviews.

Reviewer 1

This manuscript reports a qualitative study of the work lives of early-career academics with ADHD. Drawing on semi-structured interviews with 20 participants across 10 countries, the authors use a critical realist thematic analysis to examine neurotype-environment fit and friction in academic workplaces, and to consider how ADHD may matter for both individual career trajectories and collective knowledge production.

I found the manuscript engaging, timely, and potentially valuable. The topic is under-studied, the first-person accounts are rich, and the ecological framing provides a useful way to move beyond a simple deficit-versus-strengths account of ADHD. However, several points require clarification before publication, especially the strength of the collective-function claims, the transparency of the analytic process, and the implications of the sample composition.

We thank the reviewer for their positive engagement with the manuscript and the useful feedback. We think that the manuscript has improved thanks to the revisions prompted by these comments, detailed below.

1. Please moderate and clarify the claims about collective academic functioning.

One of the manuscript's most interesting contributions is the argument that ADHD-related traits may benefit collective academic functioning, for example through idea generation, interdisciplinary thinking, pattern recognition, or challenging established assumptions. This is a valuable and original angle. However, the empirical basis for this claim appears to be mainly participants' own reflections on their work styles and perceived contributions. That is appropriate for a qualitative study of first-person experience, but it does not directly demonstrate collective-level outcomes in research groups, departments, or disciplines.

I recommend distinguishing more explicitly between participants' perceptions of their contributions, the authors' theoretical interpretation of those perceptions through an ecological model, and any stronger claim about actual benefits to collective knowledge production. The manuscript would be more convincing if the collective-function argument were framed as theoretically plausible and grounded in participants' accounts, rather than as an empirically demonstrated outcome of the study.

We appreciate the reviewer's interest in our contributions regarding collective academic functioning, and we recognise that we should be more explicit about the scope of these contributions.

We have revised the Introduction, highlighting that the ecological view informs our theoretical interpretations of the data (pp. 9-10):

Thus, one of the aims of this study is to explore how ADHD academics' perspectives on their own experiences (whether strengths or challenges) at the individual level might inform theorising about ADHD contributions to academia at the collective level.

We have also revised the Discussion (with the addition of the second sentence below, (p. 40), making it more explicit that further work is needed to support our interpretations based on subjective perspectives.

To examine the contribution of cognitive diversity in knowledge production contexts more fully, different methodological approaches would be needed that harness larger-scale institutional and national data. This includes testing various contexts (institutions, academic fields, national cultures, etc.) in order to evaluate whether (and if so, when) ADHDers' potential contributions to collective academic progress—currently, reflected in participants' subjective perspectives and our theoretical interpretations thereof—actually materialise.

2. Please provide more detail on the analytic process and reflexivity.

The methods section describes the use of critical realist thematic analysis and refers to Fryer's five analytic steps. This is helpful, but the reader would benefit from more concrete detail about how the analysis moved from transcripts to codes, subthemes, and the two final themes. For example, it would be useful to clarify how initial codes were generated, how coding differences were discussed, how theoretical concepts were introduced into the coding scheme, and how disconfirming cases affected the final thematic structure.

We have added some additional details in the Method section as follows:

Codes were both semantic and latent. Subsequently, Author 1 recursively reviewed the coding scheme by collapsing similar codes, broadly grouping them in an ecological framework according to whether they related to ‘intrinsic’ features of ADHD, person-environment fit in academia, or lifespan trajectories. (p. 16)…

In the latter stages, codes were selectively aggregated in a more interpretative analysis, beginning to identify potential underlying structures (i.e., generative mechanisms, or causal tendencies, that produce events and experiences).

Initial themes were developed to capture causal tendencies that could address the research questions, accounting for the diverse experiences recounted by participants. (p. 16) …

…assessing the explanatory power of the analysis by revisiting the data, identifying disconfirming cases, and amending the thematic structure accordingly. (p.16) …

A reporting checklist, summarising these and other design decisions, appears as Supplementary Materials 1; the project’s online repository (https://osf.io/hb32d/) contains data files that track the evolution of codes from Step 3 above. (p. 17)

The additional materials linked via the project’s osf page provide concrete examples of how the coding scheme was developed, refined, and eventually mapped to themes, with illustrative data extracts for each code that contributed to the final thematic analysis.

Relatedly, the authors appropriately disclose that some members of the team are neurodivergent academics and describe reflexive practices such as journaling, team discussion, disconfirming-case analysis, and member checking. This is a strength. The paper would be stronger if it gave a brief example of how insider positionality shaped recruitment, interviewing, interpretation, or theme development.

Several decisions about study design and analysis followed directly from our insider positionality. For example, early in the project we agreed that it would be plausible if the careers of ADHD academics were more meandering than those of neurotypicals, and that we should thus err on the side of caution by having a relatively broad window for "early career". Ten years post PhD is not unusual as a threshold (cf. https://www.autism-insar.org/page/ECC), yet numerous funding bodies have a much narrower window.

A key early discussion point was whether to recruit only those with a formal diagnosis or to include those who self-identified, predicting that this might be seen as a sticking point by readers without direct experience of the diagnostic process and its various hurdles. We decided in favour of the more inclusive option as this reflected our own experiences (for those of us diagnosed later in life). Receiving a formal diagnosis of ADHD may have impacted several aspects of our lives (e.g., access to prescription medication), but we considered that our own experiences as academics prior to formal diagnosis would still have counted as relevant to this topic, given that team meetings revealed numerous shared experiences recognisable even at the ‘self-diagnosis’ stage.

The interviews themselves were enhanced by our insider status, in the sense that interviewers and interviewees often acknowledged how pleasant it was to follow various tangents and detours without fear of being judged for doing so. These shared experiences also informed our approach to analysis: there was no shortage of experiences that we recognised in ourselves when reading interview transcripts, including topics that we had previously discussed and labelled in team meetings.

Nonetheless, we were careful to check that our analyses were not simply projections of our own experiences onto participants: all team members read through the transcripts and those team members responsible for analysis revised their coding scheme numerous times in response to potential differences of perspectives. Crucially, during member checking, participants typically endorsed our interpretations (with further details on this process in the response to the next comment, including cases where we updated our interpretations as a result of participant feedback).

We have summarised key points from the above response in the following revision to the ‘Reflexivity, limitations and future directions subsection’ (pp. 38-39):

These insider positionalities influenced research design and analysis. For example, team discussions drawing on our collective experiences informed our choices to interpret "early career" quite broadly; to include self-identified ADHDers; and to allow flexibility in interview modality and questions. The wide window for “early career” means that participants may have differed in the roles and employment conditions they encountered. Regarding the choice to be more inclusive in recruitment, we note that the impact of diagnosis was not one of our research questions, but that self-identified interviewees reported similar experiences (e.g., in terms of challenges faced, perceptions of own abilities, or reflections on academic careers) as those with formal diagnosis.

More detail on member checking would also help: what materials participants received, what feedback was provided, and what kinds of amendments were incorporated.

All participants were emailed a link to the manuscript prior to submission, and they were invited to give feedback on our analyses and usage of quotations. Seven participants provided feedback.

Participant feedback was overwhelmingly positive, with respondents noting that the findings deeply resonated with their lived experiences, for instance describing a sense of validation and recognition regarding “patchwork” careers, hyperfocus and the emotional toll of academic structures.

Crucially, their feedback directly shaped the manuscript in two key ways:

- One participant's feedback highlighted how a paragraph about marking deadlines missed an important piece of context: "I did also see my quotes in there which was pleasing... I just wondered whether the paragraph before my quote on Page X might be clearer if it was more explicit as the subject was the 5-day extension for students with ADHD, but it wasn't clear that this was the student's benefit. I think the context is a little out as I was saying that if I couldn't meet the marking deadline because I was having difficulty marking because of my ADHD, that my manager would give the part I couldn't finish to another lecturer…"

Accordingly, we amended this section to highlight the disparity between formalised extensions for students versus the informal, guilt-laden redistribution of uncompleted work among colleagues for academic staff.

- Another detailed piece of feedback highlighted how burnout is frequently driven by systemic workloads as opposed to purely individual limitations: "I do think that your current description seems to focus a bit too much on the individual factors behind it ('periods of high energy led to frequent periods of burnout'), but I think it is structural factors that lead to the creation of that cycle... You spend so much time thinking 'I can't do the work because I am bad at it' that you don't realize that 'I am great at the job, but the quantity is so high that NO ONE could do all the work' is even a possibility."

We integrated this perspective into our analysis of the burnout cycle to ensure that the manuscript captures both individual experiences and systemic contexts.

We have summarised this process in the following addition to the manuscript (p. 11):

A link to the preprint was sent to all participants prior to journal submission with an invitation to feed back on our analysis, including use of their quotations. Seven participants provided feedback, which largely endorsed the analysis, and resulted in two amendments: (1) we provided more context to one data quotation; (2) we integrated the impact of systemic workloads in our analysis of the burnout cycle.

3. Please discuss the sample composition and recruitment strategy more directly.

The sample is appropriate for an exploratory qualitative study, but it is also quite specific. Participants were recruited through online academic and neurodiversity networks, were willing to discuss ADHD in an interview, were sufficiently fluent in English, and were mostly White, based in the Global North, and concentrated in psychology, education, neuroscience, biological sciences, and social sciences. These features likely shaped the themes. For example, participants connected to neurodiversity networks may be more likely to use neurodiversity-affirming language or to interpret their experiences through that framework.

The limitations section notes some of these points, but the manuscript could go further in explaining how they may have influenced the findings. The paper should avoid implying that the themes necessarily describe early-career academics with ADHD more broadly. The findings may be better framed as a detailed account of a particular, theoretically informative group of participants.

To provide more information about recruitment, we have made the following revisions to the Methods section (p. 11):

Recruitment combined features of purposive sampling (by design, we targeted early-career academics with ADHD) and convenience sampling (we recruited by advertising in our established professional networks and on social media).

Regarding the particular composition of our sample, we have added the following limitations to the Discussion (pp. 39-40):

Our recruitment strategy was necessarily limited to those with sufficient English ability to discuss these complex topics, but may additionally (and inadvertently) have selected for various features that influenced our interpretive themes, including openness to discuss topics such as diagnosis and disability; familiarity with the neurodiversity paradigm; and adult (as opposed to childhood) diagnosis. Taken together, these features likely inform participants' experiences of ADHD (including medical/support pathways) and professional life.

Similarly, recruitment through our existing professional networks and social media platforms made it easier to reach those in the Global North. Thus, our findings might not generalise to other contexts, including areas where ADHD is more stigmatised, or where professional support for disabilities is more informed by biomedical/deficit paradigms.

4. Please clarify how diagnostic status was handled analytically.

The decision to include both formal ADHD diagnosis and self-identification is understandable, especially given barriers to ADHD assessment. The manuscript states that 17 of 20 participants had a clinical diagnosis, but it is not clear whether or how diagnostic status was considered during analysis. Were the three self-identified participants' accounts broadly similar to those of diagnosed participants? Did any themes depend on diagnosis-related experiences, such as access to accommodations, disclosure, or reframing past educational experiences?

The authors do not need to separate the groups formally, and doing so may not be useful with this sample size. However, a short explanation of how diagnostic status was treated analytically would increase confidence in the interpretation.

We can confirm that the self-diagnosed participants' accounts fit well within the thematic structure we developed, and that their experiences were broadly similar to those of formally diagnosed participants. At the same time, we emphasise that the impact of diagnostic status was not among the research questions informing this particular study.

Our reasons for taking an inclusive approach to recruitment, in terms of diagnostic status, are outlined in our response to comments about insider positionality above. Reflecting on our own experiences prior to formal diagnosis (for those of the authorship team who are formally diagnosed with ADHD), we expected experiences to be broadly similar in terms of the kinds of topics probed during these interviews, across self-diagnosed and formally diagnosed individuals.

This is not to say that formal diagnosis does not bring different experiences (e.g., in terms of medication). Rather, the interviews were more concerned with what it is like to experience academia as an ADHDer and we know from first-hand experience what it is like to work in academia while medicated and while unmedicated. We acknowledge that medication is just one possible consequence of formal diagnosis, so the preceding point is just an illustration. Still, we were struck by the overlapping experiences across participants and authors, despite various differences in background and academic careers.

We note, too, that the far larger literature on autism supports the claim that self-identified and formally diagnosed individuals share numerous similar experiences, including experiences of autism (https://doi.org/10.1177/25739581251409920). Though the literature on ADHD in this regard is comparatively under-developed, we think it plausible that the same would hold true for us.

We recognise that ‘self-diagnosis’ of ADHD raises a range of questions for society and psychiatry. People, aware of inaccuracies of information online, largely do not appear to rely on social media such as TikTok to self-identify with neurodivergences like ADHD, but rather to find community (https:// doi.org/10.1177/1329878X251381388). We stress that our sample of academics do not seem to have trivially jumped to conclusions regarding their ADHD, so we do not think it crucial to engage with potential issues of overdiagnosis in this paper (which may be overblown, e.g. https://www.cambridge.org/core/journals/the-british-journal-of-psychiatry/article/adhd-over-diagnosis-fiction-fashion-and-failure/1163426C23804A7049FE35D940EA938C). Finally, we note that 17 of our 20 participants did have a clinical diagnosis of ADHD.

We have summarised key points from the preceding discussion in the following revision (p. 39):

Regarding the choice to be more inclusive in recruitment, we note that the impact of diagnosis was not one of our research questions, but that self-identified interviewees reported similar experiences (e.g., in terms of challenges faced, perceptions of own abilities, or reflections on academic careers) as those with formal diagnosis.

Minor comments

1. The abstract is informative but dense. Consider simplifying the description of the ecological contribution so that the central research question and main findings are easier to grasp for non-specialist readers.

We have streamlined the relevant passage as follows (p. 2):

It also highlights an ecological view by exploring how ADHD matters both for individual careers and for collective knowledge production.

2. The terms "function", "functioning", "collective functioning", and "academic functioning" are central to the manuscript. A concise definition early in the paper would help readers follow the argument.

Thank you for this suggestion. In our revision, we have highlighted the polysemous use of "functioning" as follows (p. 6):

First, though, we note that the term "functioning" will occur in several senses here. These are conceptually distinguishable but frequently overlap in our interpretation of participants' experiences, hence our deliberately holistic approach to this term. Depending on context, it can variously refer to:

● Cognitive functioning: differences in basic cognitive processes including executive function, attention, and memory.

● Everyday functioning: differences in managing or navigating the demands of daily life (often—but not necessarily—related to cognitive functioning) with implications for subjective wellbeing.

● Professional functioning: differences in cognitive/everyday functioning applied to a work setting (here, an academic setting, with implications for research productivity, teaching, and other professional activities)

● Collective functioning: broader group-level patterns in scientific progress, where this is conceived of as an inherently social enterprise with sometimes-counterintuitive mappings between individual performance and collective outcomes

3. The discussion would benefit from a short, concrete statement of practical implications for universities, such as accessible administrative processes, supervisor training, workload management, or accommodation processes co-designed with neurodivergent academics.

As the reviewer mentioned above, our recruitment strategy yielded specific features in the sample that limit generalisability. We thus think it premature to make a concrete statement regarding specific implications for universities. Still, we take the reviewer's point that some lessons for policy can be drawn from our findings, though we have chosen to frame these as "ways to think about policy improvement", rather than actual new policies. The following is a new subsection of the Discussion setting out these points (pp. 37-38).

Towards practical recommendations

Given the targeted nature of our sample and the interpretive nature of our analysis, we cannot yet propose concrete recommendations for universities and administrators to support ADHDers in academia. Nonetheless, our findings highlight useful ways to think about relevant issues, informing how future work might develop such recommendations.

First, organisational procedures and policies should bear in mind tensions and synergies identified above, and should thus prioritise thinking holistically about solutions. For example, we described how flexibility is appealing to ADHDers, but also how this clashes with the unbounded nature of academic work, risking juggle/crash cycles. Thus, plans for policy development should not aim at one such dimension (e.g., enhancing flexibility, for instance via "job crafting") without also considering these tensions (e.g., managing unboundedness). Conversely, we described how novelty is a common drive for ADHDers. Thus, policies aimed at supporting flexible working should also be evaluated in terms of how they allow flexibility to develop key motivations such as novelty.

Second, mentors and managers (or other colleagues) should be aware of the dynamic shifts described here. For example, we described how ADHDers may experience periods of energetic productivity followed by periods of burnout. These periods also had implications for ADHDers' wellbeing and their estimations of their own performance. Plans for policy development should thus equip mentors and managers to support non-linear energy rhythms, rather than to expect continuous output. Similarly, collaborators might consider how their own contributions can complement ADHD working styles, for instance by choosing to support task prioritisation, task initiation, and flexible milestone tracking aimed at project completion. A related challenge is to recognise these dynamic shifts in the first place, given patterns of masking and effortful staying between the lines described above.

Finally, institutional policies (e.g., for hiring or promotion) should be aware of how ADHD career trajectories might look different from typical trajectories. For instance, we described how ADHDers might prefer to explore widely, connecting currently unrelated methods or problems. While these pluralistic, big-picture approaches can benefit science, they are inherently risky for individual researchers. Similarly, the patterns described here (deep dives, side quests or going down rabbit holes) will not necessarily yield the usual signals of productivity in academia (e.g., number of papers published). Policies that aim to be inclusive should also rethink evaluation in qualitative, not just quantitative terms. Doing so could articulate explicit roles for neurodiversity in meeting calls for "slow science" [63, 64].

Overall, aspirations to be more inclusive of ADHDers in academia should incorporate ADHD lived experiences into the policy development process, including via further qualitative research on those experiences.

4. The paper defines early-career academics as including PhD students through 10 years post-PhD. Because this is a broad span with different institutional power and employment conditions, the authors could acknowledge that "early career" may encompass quite different workplace experiences.

Our choice of a 10-year threshold is addressed in our response regarding insider positionality above, and we have further revised the Discussion to acknowledge this issue (p. 39):

The wide window for “early career” means that participants may have differed in the roles and employment conditions they encountered.

Reviewer 2

It is a pleasure to review a clearly written manuscript on such an interesting and important topic. The manuscript has several strengths, including an explicit discussion of reflexivity, a clearly stated critical realist approach, and a careful, well-designed interview process that takes participants’ language and communication preferences into account. However, some points would benefit from further clarification and justification. As noted below, I suggest consulting the COREQ and/or SRQR reporting guidelines and, if appropriate, including a completed checklist as supplementary material to improve the methodological reporting of the manuscript.

We thank the reviewer for their positive engagement with the manuscript and the useful feedback. We think that the manuscript has improved thanks to the revisions prompted by these comments, detailed below.

Major suggestions

1. Strengthen COREQ-aligned reporting

Several important COREQ items are missing or only partially reported. It would be useful to explicitly state which authors conducted the interviews; the interviewers’ credentials, occupations, gender, and relevant experience or training; whether any prior relationship existed with participants; what participants knew about the interviewers’ positionality or reasons for conducting the study; whether non-participants were present; whether repeat interviews occurred; whether field notes were taken; whether transcripts were returned to participants; what software, if any, was used; and whether there were any cases of non-participation, refusal, ineligibility, or dropout. A supplementary COREQ table would address this efficiently.

Thank you for the suggestion. We have added Supplemental Material 1 (SM1) which includes COREQ-relevant information.

2. Clarify the conceptual status of ADHD in the manuscript

From a conceptual perspective, the manuscript would benefit from a clearer account of how ADHD is framed by the authors. The different ways in which ADHD is referred to may create confusion regarding whether it is considered a form of psychopathology, a neurodevelopmental condition, a disability, a non-pathologising identity category, or some combination of these frameworks. This is understandable given the topic, but the authors’ conceptual position should be clearer. This is especially important because the analysis makes claims about “ADHD neurotype” and “causal tendencies.” I suggest adding a short paragraph explaining how the authors understand ADHD in relation to diagnostic criteria, self-identification, neurodiversity-informed language, and psychopathology/disability frameworks.

Thank you for this suggestion. We have revised the text to make it more explicit how our conceptualisation of ADHD is consistent with the neurodiversity paradigm rather than rooted in a biomedical paradigm focused on psychopathology (pp. 4-5):

There are diverse, sometimes conflicting conceptualisations of ADHD [5]. ADHD is clinically defined in terms of inattention, hyperactivity, and impulsivity symptoms. Here, we adopt a conceptualisation more consistent with the neurodiversity paradigm: framing ADHD in terms of "difference" rather than "deficit"; resisting purely neurological accounts by highlighting personal experiences (which often reflect brain/biological differences without always being fully reducible to those); expecting these experiences to be somewhat fluid across contexts; highlighting strengths as well as challenges; and situating both strengths and challenges (including disability) in person–environment interactions. Accordingly, by "ADHD neurotype," we mean both cognitive traits and subjective experiences that are common among ADHDers (including cognitive dynamism, adventurousness, and the ability to hyperfocus on topics of interest [6]), without assuming that there is a necessary and sufficient set of these traits or experiences that must be universal among (or unique to) ADHDers.

3. Justify the inclusion of self-identified ADHD participants more explicitly

The manuscript states that formal diagnosis and self-identification were equally acceptable because of barriers to clinical ADHD assessment. This is defensible, but it requires further methodological justification. This point also relates to the previous comment and could be clarified in the same section.

Additional reasons for taking an inclusive approach to recruitment, in terms of diagnostic status, are grounded in our insider positionality. Reflecting on our own experiences prior to formal diagnosis (for those of the authorship team who are formally diagnosed with ADHD), we expected experiences to be broadly similar in terms of the kinds of topics probed during these interviews, across self-diagnosed and formally diagnosed individuals.

We recognise that self-diagnosis of ADHD raises a range of questions for society and psychiatry (e.g., due to its prevalence on social media: https://doi.org/10.1080/02667363.2024.2409451), but we stress that our sample of academics do not seem to have trivially jumped to conclusions regarding their ADHD, so we do not think it crucial to engage with potential issues of overdiagnosis in this paper.

We have addressed these discussion points in the following revision (p. 39):

Regarding the choice to be more inclusive in recruitment, we note that the impact of diagnosis was not one of our research questions, but that self-identified interviewees reported similar experiences (e.g., in terms of challenges faced, perceptions of own abilities, or reflections on academic careers) as those with formal diagnosis.

4. Clarify the sampling strategy and sample adequacy

The inclusion criteria are partly described, but the sampling strategy should be named more explicitly, whether purposive sampling, convenience sampling, snowball sampling, or another strategy. The authors should also explain how the final sample size was judged adequate. The current manuscript does not make clear why 20 interviews were sufficient for the analytic claims being made.

We have revised the manuscript as follows (p. 11):

Recruitment combined features of purposive sampling (by design, we targeted early-career academics with ADHD) and convenience sampling (we recruited by advertising in our established professional networks and on social media).

Regarding sample size, we have added the following as footnote 1, where "SM1" points to the requested reporting of COREQ-relevant information.

We note that "sample size justification" is an ill-defined term in qualitative research, and that we judge the adequacy of our data by inherently subjective features such as richness and depth. For further discussion, see SM1.

5. Make the critical realist analytic process more transparent

The use of critical realist thematic analysis is a strength, but some key analytic terms remain abstract. The manuscript would benefit from a clearer explanation of how the authors moved from participant accounts to codes, then to events, causal tendencies, and explanatory themes. Terms such as “retroductive reasoning” and “causal tendencies” should be briefly operationalised for the reader. A short analytic example, coding tree, or supplementary analytic trail would strengthen the credibility of the analysis.

Thank you for this suggestion. First, we have added several data files to the project's repository at the Open Science Framework (https://osf.io/hb32d). These track the evolution of codes across several stages of analysis. Second, we have updated the main text as follows:

(footnote 2) "Retroductive reasoning" is the process of working back from data to generate potential explanations for those data.

We have moved the definition of "Causal tendencies" forward, so that the definition now occurs next to its first usage in the manuscript (p. 15):

‘Causal tendency’ refers to the potential for a factor to exert an effect, based on its own properties and its relations with other factors.

6. Moderate or further support claims about collective academic functioning

The findings concerning individual experiences, institutional barriers, masking, overcommitment, burnout, and career trajectories are well supported by participant quotations. The claims about collective-level benefits to knowledge production are plausible and theoretically interesting, but they are more inferential. The authors should more clearly distinguish between participant-reported experience, critical realist interpretation, and theoretical extrapolation. Some statements about ADHD academics benefiting knowledge production could be phrased more cautiously unless additional analytic support is provided.

We appreciate the suggestion to be more explicit about the scope of our contribution regarding collective functioning. Accordingly, we have revised the Introduction, highlighting that the ecological view informs our interpretation of the data and is primarily theoretical (pp. 9-10 ):

Thus, one of the aims of this study is to explore how ADHD academics' perspectives on their own experiences (whether strengths or challenges) at the individual level might inform theorising about ADHD contributions to academia at the collective level.

We have also revised the Discussion (with the addition of the second sentence below), making it more explicit that further work is needed to support our interpretations based on subjective perspectives (p. 40).

To examine the contribution of cognitive diversity in knowledge production contexts more fully, different methodological approaches would be needed that harness larger-scale institutional and national data. This includes testing various contexts (institutions, academic fields, national cultures, etc.) in order to evaluate whether (and if so, when) ADHDers' potential contributions to collective academic progress—currently, reflected in participants' subjective perspectives and our theoretical interpretations thereof—actually materialise.

7. Expand the limitations and transferability discussion

The authors appropriately state that the study does not aim for generalisability, but the transferability discussion should be more specific. In addition, the mean age of diagnosis suggests that the sample largely reflects late-diagnosed adults, which may shape retrospective self-understanding and workplace narratives. These issues can be discussed as limitations to transferability.

We have substantially revised the Discussion to be more specific about these points (including the comment about late-diagnosed adults) (p. 39-40):

Our recruitment strategy was necessarily limited to those with sufficient English ability to discuss these complex topics, but may additionally (and inadvertently) have selected for various features that influenced our interpretive themes, including openness to discuss topics such as diagnosis and disability; familiarity with the neurodiversity paradigm; and adult (as opposed to childhood) diagnosis. Taken together, these features likely inform participants' experiences of ADHD (including medical/support pathways) and professional life. Similarly, recruitment through our existing professional networks and social media platforms made it easier to reach those in the Global North. Thus, our findings might not generalise to other contexts, including areas where ADHD is more stigmatised, or where professional support for disabilities is more informed by biomedical/deficit paradigms.

Minor comments

1. Member checking should be described in slightly more detail.

The use of member checking is a strength. Please clarify how many participants were invited, how feedback was collected, and whether any feedback challenged or changed the thematic structure.

All participants were emailed a link to the manuscript prior to submission, and they were invited to give feedback on our analyses and usage of quotations. Seven participants provided feedback.

Participant feedback was overwhelmingly positive, with respondents noting that the findings deeply resonated with their lived experiences, for instance describing a sense of validation and recognition regarding “patchwork” careers, hyperfocus and the emotional toll of academic structures.

There were two instances of pushback, which directly shaped the manuscript:

- One participant's feedback highlighted how a paragraph about marking deadlines missed an important piece of context: "I did also see my quotes in there which was pleasing... I just wondered whether the paragraph before my quote on Page X might be clearer if it was more explicit as the subject was the 5-day extension for students with ADHD, but it wasn't clear that this was the student's benefit. I think the context is a little out as I was saying that if I couldn't meet the marking deadline because I was having difficulty marking because of my ADHD, that my manager would give the part I couldn't finish to another lecturer…"

Accordingly, we amended this section to highlight the disparity between formalized extensions for students versus the informal, guilt-laden redistribution of uncompleted work among colleagues for academic staff.

- Another detailed piece of feedback highlighted how burnout is frequently driven by systemic workloads as opposed to purely individual limitations: "I do think that your current description seems to focus a bit too much on the individual factors behind it ('periods of high energy led to frequent periods of burnout'), but I think it is structural factors that lead to the creation of that cycle... You spend so much time thinking 'I can't do the work because I am bad at it' that you don't realize that 'I am great at the job, but the quantity is so high that NO ONE could do all the work' is even a possibility."

We integrated this perspective into our analysis of the burnout cycle to ensure that the manuscript captures both individual experiences and systemic contexts.

We have summarised this process in the following addition to the manuscript (p. 11):

A link to the preprint was sent to all participants prior to journal submission with an invitation to feed back on our analysis, including use of their quotations. Seven participants provided feedback, which largely endorsed the analysis, and resulted in two amendments: (1) we provided more context to one data quotation; (2) we integrated the impact of systemic workloads in our analysis of the burnout cycle.

2. Consider moving or expanding some reflexivity material in the Methods.

The reflexivity section is useful, but more COREQ-relevant information should appear in the Methods or supplementary material, particularly interviewer characteristics and researcher–participant relationships.

Thank you for the suggestion. COREQ-relevant information now appears as Supplemental Material 1 (SM1), though we briefly outline in that material why we do not exactly reproduce the standard COREQ format (a table/checklist format).

3. Provide the interview guide or sample questions.

The manuscript describes the interview schedule, but including the full guide or sample questions as supplementary material would improve transparency.

We have made the interview schedule (as well as pre-interview questionnaire) publicly available in the project's repository at the Open Science Framework (https://osf.io/hb32d/), and have added this link to the manuscript.

4. Clarify the role of autoethnographic literature in the Discussion.

When comparing the findings with autoethnographic accounts, the authors should clarify that these sources are being used as contextual or interpretive literature, rather than as direct corroborative evidence equivalent to empirical workplace studies.

We appreciate the importance of accuracy and clarity in using such terms, but for the sake of brevity (especially as the autoethnographic nature of these accounts is incidental to the point we are trying to make here), we have deleted mention of this, and have instead highlighted the relevant points as follows (p. 34):

Individual traits identified by our participants as conducive to academic work mirror ADHD workplace strengths identified in previous literature, including subjective perspectives of academics (though these comparisons are necessarily open to interpretation).

Overall, this is a promising and carefully conducted qualitative study. Addressing the points above would substantially strengthen the manuscript’s credibility, alignment with qualitative reporting standards, and interpretive clarity.

We once again thank the reviewer for their helpful input, and hope that the above revisions have addressed these comments.

Attachment Submitted filename: Response to reviewers.docx

# Editor report: Decision Letter 1

**DOI:** 10.1371/journal.pone.0361208.r003

**Authors:** Branko Aleksic

8 Oct 2026

“The significance of context”: Fit, friction, and function in the work lives of early career academics with ADHD.

PONE-D-26-26250R1

Dear Dr. Hamilton,

We’re pleased to inform you that your manuscript has been judged scientifically suitable for publication and will be formally accepted for publication once it meets all outstanding technical requirements.

Within one week, you’ll receive an e-mail detailing the required amendments. When these have been addressed, you’ll receive a formal acceptance letter and your manuscript will be scheduled for publication.

An invoice will be generated when your article is formally accepted. Please note, if your institution has a publishing partnership with PLOS and your article meets the relevant criteria, all or part of your publication costs will be covered. Please make sure your user information is up-to-date by logging into Editorial Manager at Editorial Manager® and clicking the ‘Update My Information' link at the top of the page. For questions related to billing, please contact billing support.

If your institution or institutions have a press office, please notify them about your upcoming paper to help maximize its impact. If they’ll be preparing press materials, please inform our press team as soon as possible -- no later than 48 hours after receiving the formal acceptance. Your manuscript will remain under strict press embargo until 2 pm Eastern Time on the date of publication. For more information, please contact onepress@plos.org.

Kind regards,

Branko Aleksic, MD, PhD

Academic Editor

PLOS One

Additional Editor Comments (optional):

Reviewers' comments:

# Editor report: Acceptance letter

**DOI:** 10.1371/journal.pone.0361208.r004

**Authors:** Branko Aleksic

PONE-D-26-26250R1

PLOS One

Dear Dr. Hamilton,

I'm pleased to inform you that your manuscript has been deemed suitable for publication in PLOS One. Congratulations! Your manuscript is now being handed over to our production team.

At this stage, our production department will prepare your paper for publication. This includes ensuring the following:

* All references, tables, and figures are properly cited

* All relevant supporting information is included in the manuscript submission,

* There are no issues that prevent the paper from being properly typeset

You will receive further instructions from the production team, including instructions on how to review your proof when it is ready. Please keep in mind that we are working through a large volume of accepted articles, so please give us a few days to review your paper and let you know the next and final steps.

Lastly, if your institution or institutions have a press office, please let them know about your upcoming paper now to help maximize its impact. If they'll be preparing press materials, please inform our press team within the next 48 hours. Your manuscript will remain under strict press embargo until 2 pm Eastern Time on the date of publication. For more information, please contact onepress@plos.org.

You will receive an invoice from PLOS for your publication fee after your manuscript has reached the completed accept phase. If you receive an email requesting payment before acceptance or for any other service, this may be a phishing scheme. Learn how to identify phishing emails and protect your accounts at https://explore.plos.org/phishing.

If we can help with anything else, please email us at customercare@plos.org.

Thank you for submitting your work to PLOS One and supporting open access.

Kind regards,

PLOS One Editorial Office Staff

on behalf of

Dr Branko Aleksic

Academic Editor

PLOS One
